# Zephycandidine A and Synthetic Analogues—Synthesis and Evaluation of Biological Activity

**DOI:** 10.3390/molecules30030752

**Published:** 2025-02-06

**Authors:** Thomas Klaßmüller, Florian Lengauer, Julia Blenninger, Franz Geisslinger, Karin Bartel, Franz Bracher

**Affiliations:** Department of Pharmacy—Center for Drug Research, Ludwig-Maximilians University, 81377 Munich, Germanykarin.bartel@cup.uni-muenchen.de (K.B.)

**Keywords:** zephycandidine A, alkaloid, total synthesis, structure–activity relationship, leukemia, cell death, mitochondria

## Abstract

A convenient total synthesis of the imidazo[1,2-f]phenanthridine-type Amaryllidaceae alkaloid zephycandidine A (**3**) was developed, which further allowed us to perform modifications of substituents on benzenoid ring A and imidazole ring D. The biological activities of all synthesized compounds were evaluated, and it was reported that activities against cancer cells of the parent alkaloid were poorly reproducible, while the closely related analogue THK-121 (**11**) showed a strong inhibitory effect on proliferation. Additionally, our novel analogue significantly induced cell death via the intrinsic apoptosis pathway, evident by the loss of mitochondrial membrane potential, increased mitochondrial oxidative stress, and disrupted mitochondrial structure in the same cells. At the same time, healthy cells were less affected by the treatment with THK-121 (**11**), indicating a potential therapeutic margin.

## 1. Introduction

**Figure 1:**
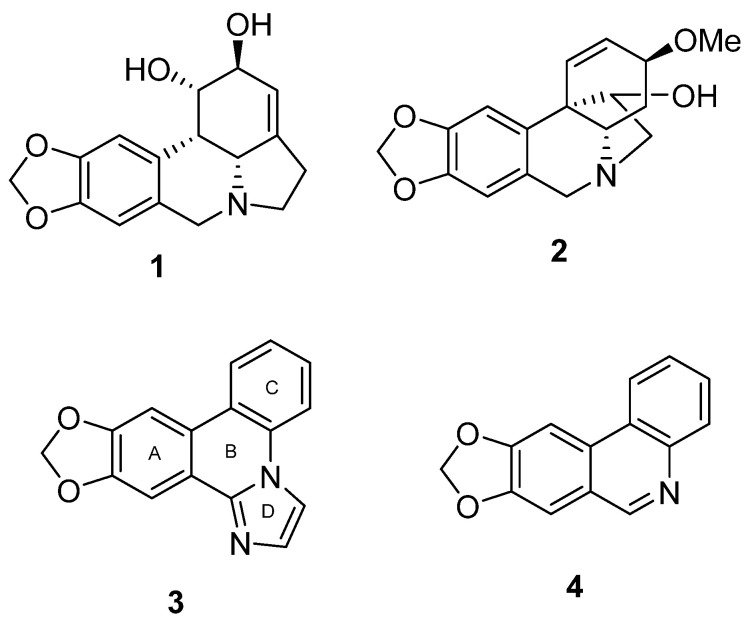
Phenanthridine-type alkaloids from Amaryllidaceae: lycorine (**1**), haemanthamine (**2**), and zephycandidine A (**3**). Phenanthridine building block **4** for the first partial synthesis of zephycandidine A (**3**).

Amaryllidaceae, a family of mainly perennial bulbous flowering plants with more than one thousand species found in tropical and subtropical regions of the world, are a rich source of bioactive alkaloids. Over decades, hundreds of alkaloids from diverse frameworks possessing acetylcholinesterase inhibitory, antimicrobial, antiviral, antitumor, and other activities have been isolated [1]. Numerous types of Amaryllidaceae alkaloids contain modified phenanthrene partial structures, among them being the lycorine (**1**) subtype with the pyrrolo[*de*]phenanthidine skeleton and the crinine subtype (e.g., haemanthamine (**2**)) incorporating the 5,10b-ethanophenanthridine skeleton (Figure 1).

In 2016, Zhan et al. [2] reported on the isolation of the first imidazo[1,2-*f*]phenanthridine-type alkaloid, zephycandidine A (**3**), from *Zephyranthes candida* (Lindl.) (Amaryllidaceae), a bulbous herb, which is cultured as an ornamental flower, and the whole plant is used in folk medicine in China. Structurally distinct alkaloids from the same plant were named zephycandidine I, II, and III in 2017 [3].

The novel new minor alkaloid zephycandidine A (**3**) was described to exhibit significant cytotoxicity against five cancer cell lines with IC_50_ values ranging from 2 to 7 μM and to induce apoptosis in leukemia cells. Furthermore, the authors observed acetylcholinesterase inhibitory activity, but this activity could not be confirmed by the Banwell group in subsequent investigations on synthetic zephycandidine A [4].

The first partial synthesis of zephycandidine A (**3**) was accomplished by Murphy et al. [5] in three steps from the alkaloid haemanthamine (**2**). The thermal degradation of **2** yielded phenanthrene **4** (Figure 1) in a poor yield, and subsequent treatment with 1,2-dibromoethane, liquid ammonia, and MnO_2_ following the annulation protocol by Cronin [6] yielded the target compound. Recently, Banwell [4] reported on two distinct novel approaches to zephycandidine A (**3**). The first method included the Pd-catalyzed cyclization of 2-arylimidazole and 1,2-dihalobenzenes through a two-fold cross-coupling process, which afforded a poorly separable mixture of two isomeric products; the second route resulted in a lower yield (32% in the final step), where regioselectivity was achieved by the introduction of an additional bromo substituent and the utilization of expensive 2-iodophenylboronic acid, yielding zephycandidine A (**3**). Recently, another total synthesis process involving going through bromoaryl-imidazole and utilizing a non-aromatic ring C building block (1,3-cyclohexanedione) with limited variability and a complex catalyst system was published by Lee et al. [7]. Two other general approaches to the imidazo[1,2-f]phenanthridine backbone (not yet applied to the total synthesis of zephycandidine A) utilize readily available 6-aminophenanthridine building blocks. The attachment of the 2,3-unsubstituted imidazole ring is conveniently achieved by treatment with chloroacetaldehyde [8,9], 3-substituted analogues are produced from 6-aminophenanthridines, and aliphatic aldehydes/arylacetaldehydes are produced through a novel, metal-free sulfur endorsed oxidative cyclization protocol developed by Zhang’s group [10]. Furthermore, 6-chlorophenanthridine can be condensed with aminoacetaldehyde dimethylacetal to yield 2,3-unsubstituted imidazo[1,2-f]phenanthridines [11].

The reported antitumor activity of zephycandidine A (**3**) from the plant source prompted Zhang’s group to perform studies on the activity of synthetic analogues they obtained in the course of the elucidation of the scope of their oxidative cyclization protocol [10]. The collection of test compounds was characterized by diverse substituents at C-3 (phenyl, benzyl, and linear and branched alkyl residues); furthermore, a few substituents were introduced on the benzenoid rings. Among these test substances, 3-methyl- and the 3-*tert*-butyl imidazo[1,2-f]phenanthridines showed marked inhibitory activity against several tumour cell lines (top values about 2 µM). Unfortunately, zephycandidine A (**3**) was not included in this substance library, and neither was the 9a,12a-methylenedioxy unit, a privileged structure motif in bioactive natural products, and similar oxygenation patterns (except one monomethoxy analogue) were not present among the investigated compounds. This prompted us to investigate the cytotoxic activities of (a) methylenedioxy-decorated imidazo[1,2-f]phenanthridines bearing additional substituents at the imidazole ring (C-3), as well as (b) analogues with a modified oxygenation pattern at ring A and one congener with a pyridine as ring A in a panel of different cancer cell lines.

## 2. Results and Discussion

### 2.1. Chemistry

**Scheme 1:**
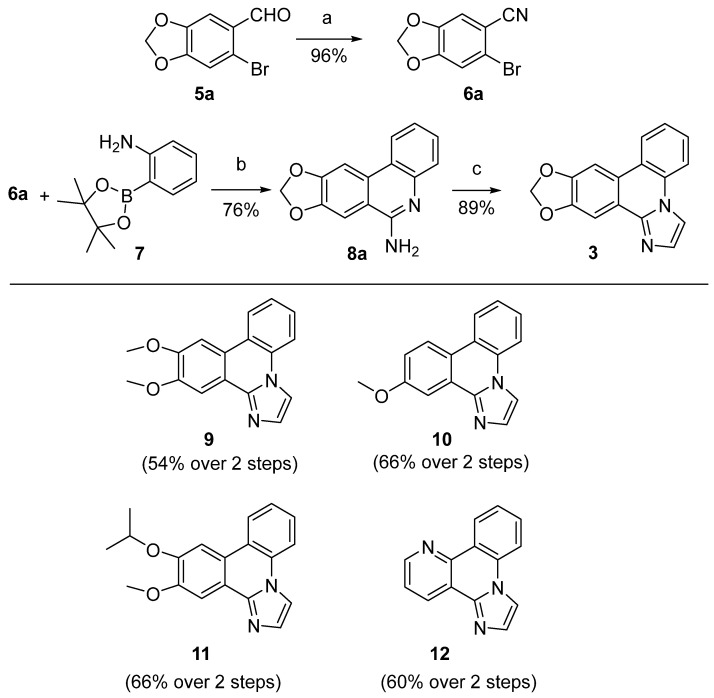
The general route for the synthesis of zephycandidine A (**3**) and ring A-modified analogues (**10**–**12**) synthesized following the same protocol via the corresponding (hetero)aromatic *ortho*-bromonitriles **6**. Reagents and conditions: (a) H_2_NOH-HCl, DMSO, 90 °C, 1 h; (b) cat. PdCl_2_(Ph_3_P)_2_, DMF, aq. Na_2_CO_3_, 80 °C, 16 h; (c) chloroacetaldehyde, Na_2_CO_3_, 2-propanol-water, reflux, 1 h.

Zephycandidine A (**3**) and analogues with an unsubstituted imidazole ring (ring D) and with modifications in ring A were synthesized by adapting the approach of Zhang et al. [8]. A new and effective total synthesis method for zephycandidine A (**3**) was developed starting from commercially available building block 6-bromo-1,3-benzodioxole-5-carbonitrile (**6a**), which is alternatively prepared in one step from cheap 6-bromopiperonal (**5a**) by oximation/dehydration [12] in a 96% yield. The Suzuki–Miyaura cross-coupling of **6a** and 2-(4,4,5,5-tetramethyl-1,3,2-dioxaborolan-2-yl)aniline (**7**) gave, after spontaneous intramolecular nucleophilic amine-to-nitrile addition, 6-aminophenanthridine **8a** in a 76% yield. A subsequent reaction with chloroacetaldehyde and Na_2_CO_3_ in isopropanol/water gave alkaloid **3** in a 89% yield (Scheme 1). In accordance with reports from other groups [4,5], the alkaloid was obtained as a high-melting solid, whereas Zhan et al. [2] surprisingly described that their compound was isolated from the plant source as a colourless oil. Notably, Zhan’s published ^1^H and ^13^C NMR spectra are fragmentary (in the ^1^H NMR spectrum, the part below 5.4 ppm is missing, and in the ^13^C NMR spectrum, the part below 54 ppm is missing); thus, the presence of eventually bioactive (see the discussion below) aliphatic impurities in the isolated natural product cannot be excluded. Our two-step procedure (68% overall yield) represents the most effective total synthesis of zephycandidine A at present.

This reaction sequence can easily be extended to the synthesis of zephycandidine A analogues with modified substitution patterns in ring A (methoxy and isopropoxy residues) since the required *ortho*-bromobenzonitriles **6b**–**d** (for details, see the Experimental Procedure Section) were easily accessible from easy-to-prepare or, optionally, commercially available *ortho*-bromobenzaldehydes **5b**–**d** via conversion to oximes and concomitant dehydration in hot DMSO [12]. The benzodioxole ring system was replaced by a pyridine ring (compound **12**) in the same manner by starting with 2-bromopyridine-3-carboxaldehyde (**5e**). The target compounds **9**–**12** obtained this way are shown in Scheme 1.

**Scheme 2:**
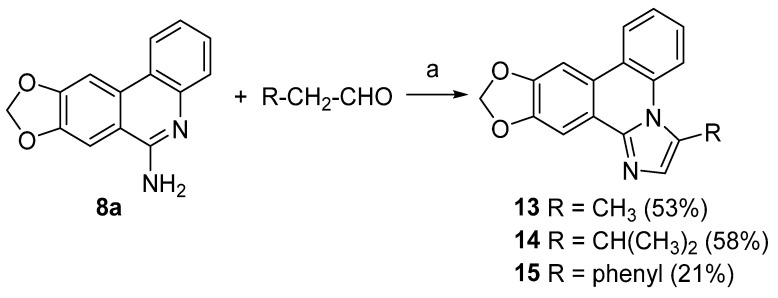
Sulfur-endorsed construction of substituted ring D yielding products **13**–**15**. Reagents and conditions: (a) sulfur, DMSO/cyclohexane, 120 °C.

For the synthesis of analogues of zephycandidine A (**3**) bearing additional substituents at imidazole ring D (C-3) (pertaining the methylenedioxy motif at ring A), we utilized intermediate **8a** from the zephycandidine A synthesis process (Scheme 1). A reaction with appropriate aldehydes (phenylacetaldehyde, propanal, and 3-methylbutanal) in DMSO/cyclohexane with excess elemental sulfur at 120 °C [10] gave the desired products **13**–**15** in moderate yields (21–58%) (Scheme 2).

The obtained library of zephycandidine A (**3**) and seven analogues were subjected to an analysis of their biological activity against several cancer cell lines.

### 2.2. Evaluation of Biological Activity

**Figure 2:**
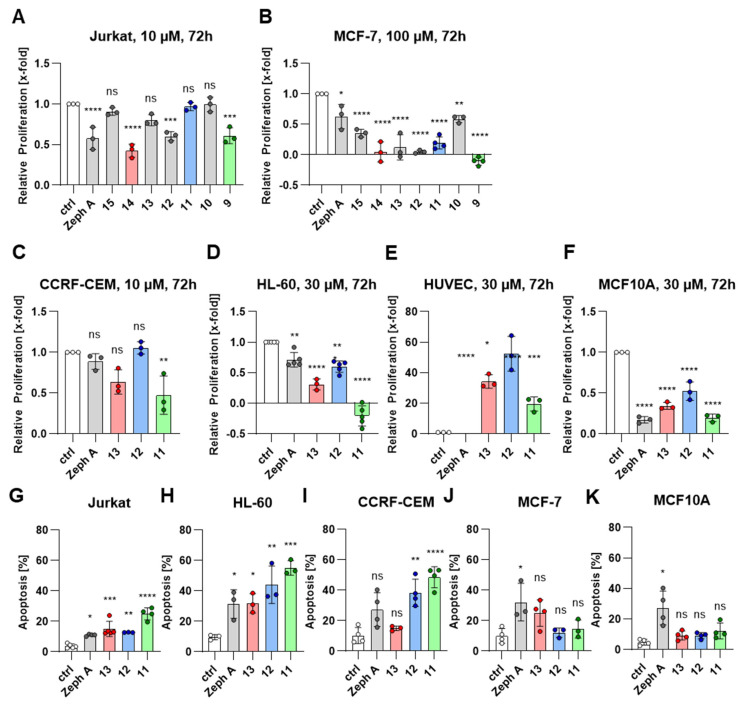
Zephycandidine A analogues decrease proliferation and induce cell death. (**A**–**E**) Proliferation was determined in Jurkat (**A**), MCF-7 (**B**), CCRF-CEM (**C**), HL-60 (**D**), HUVEC (**E**), and MCF10A (**F**) cells by CellTiter^®^-Blue cell viability assay. Cells were treated as indicated for 72 h. Data are shown as mean ± SD (*n* = 3). (**F**–**I**) Cell death at IC_50_ values determined in (**A**–**E**) was assessed after 48 h by forward versus sideward (FSC vs. SSC) scatter gating for Jurkat (**G**), HL-60 (**H**), and CCRF-CEM (**I**) cells and by Nicoletti assay for MCF-7 (**J**) and MCF10A (**K**). Bar graphs display mean ± SD (*n* = 3). Circles indicate individual data points. One-way ANOVA was conducted, followed by Tukey’s multiple comparison test, referring to control. * *p* < 0.05, ** *p* < 0.005, *** *p* < 0.001, **** *p* < 0.0001 Graphics were in part taken (and modified) from PhD thesis of F.L.

Since accumulating evidence demonstrated promising antitumoral effects of alkaloids derived from Amaryllidaceae plants [13,14,15,16], we elucidated the potential of zephycandidine A (**3**) and our novel analogues to inhibit the growth and survival of cancer cells. First, we tested their biological activity in a CellTiter^®^-Blue proliferation assay on various cancer cell lines. Initially, we tested seven analogues on Jurkat (leukemia) (Figure 2A) and MCF-7 (breast cancer) (Figure 2B) cells, which resulted in IC_50_ values in the µM range (11.7 µM and 145 µM, respectively, for zephycandidine A (**3**); 9.6 µM to 120 µM for the synthetic analogues) for the inhibition of cancer cell proliferation (for details, see Supplementary Materials, Table S1). From these tested analogues, we chose three active compounds to characterize further: mixed diether **11** (internal code: THK-121), pyridine analogue **12**, and methylimidazole **13**. These synthetic compounds showed superior activity compared to zephycandidine A (**3**) in decreasing cell proliferation in MCF-7 and were at least equipotent in inhibiting Jurkat cell proliferation. An analysis of their anti-proliferative capacity in CCRF-CEM (Figure 2C), HL-60 (Figure 2D), and healthy endothelial HUVEC (Figure 2E) and healthy epithelial MCF10A (Figure 2F) cells also led to IC_50_ values in the µM range (Supplementary Materials, Table S1). These results are, in general, in accordance with a previous report by Zhan et al. showing that zephycandidine A had anti-proliferative potency in the low µM range against several cancer cell lines [2]. Like Zhan et al., who reported the selectivity of zephycandidine A for cancer cells over human bronchial epithelial Beas-2B cells, we found increased anti-proliferative potential of the lead structure as well as analogues against cancer cell lines compared to non-cancerous HUVEC cells. For healthy MCF10A cells, zephycandidine A reduced proliferation drastically, while the novel structures had less inhibitory effects (Figure 2F). This indicates some degree of cancer selectivity and superiority of the novel analogues. Of note, the IC_50_ values obtained for zephycandidine A in MCF7 and HL-60 cells were up to 100 fold higher in our experimental setup compared to the values reported by Zhan et al. This discrepancy might be attributed to either differences in the assays used or the origin of zephycandidine A. While Zhan et al. isolated zephycandidine A from *Zephyranthes candida* [2], we used our very pure synthesis product. As shown above, the published NMR data for the isolated alkaloid do not exclude aliphatic impurities, so we (in accordance with Banwell and co-workers [4], who could not reproduce the claimed acetylcholinesterase-inhibitory activity) suppose that the reported biological activities of the alkaloid were falsified by undetected biologically active impurities.

Nevertheless, zephycandidine A (**3**) and, even to a higher extent, synthetic analogues exhibit the potential to inhibit cancer cell growth in the low µM range and exhibit a more pronounced effect on cancer proliferation compared to healthy cells.

Next, we tested zephycandidine A and selected analogues according to their potential to induce cell death in cancer cells. Based on the identified antiproliferative activity, we used concentrations at the respective IC_50_ values and analyzed cell death using flow cytometry. The lead structure induced moderate cell death in all tested lines (Figure 2G–J), whereas the tested analogues showed an increased potential to induce cell death in leukemic cells (Figure 2G–I). In healthy epithelial MCF10A cells, only zephycandidine A induced apoptosis, while the novel analogues had no significant cytotoxic effect (Figure 2K).

**Figure 3:**
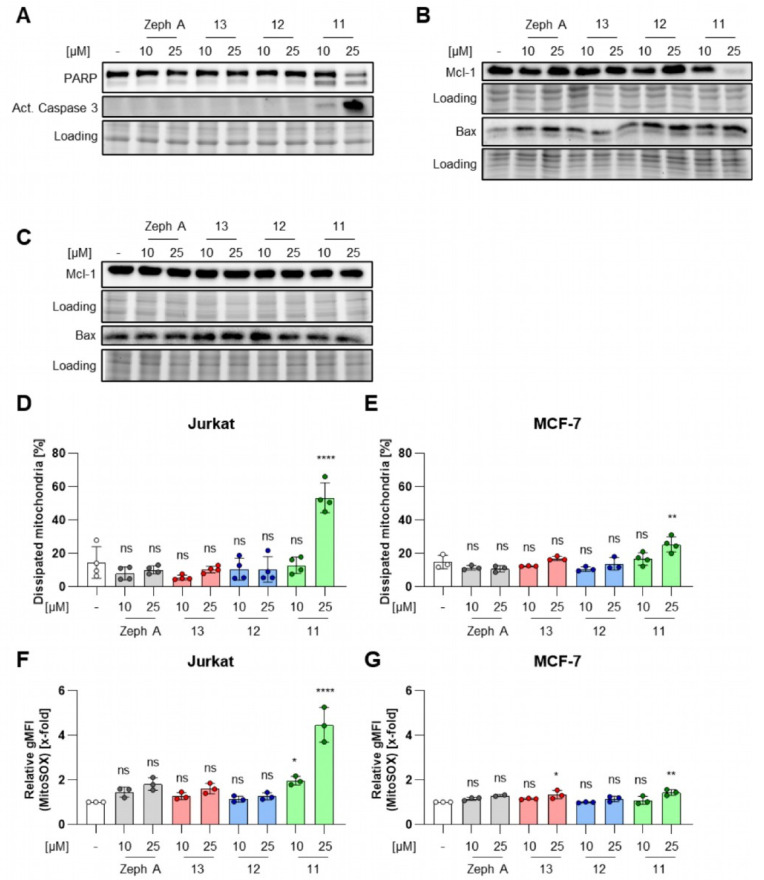
Zephycandidine A analogues induce the mitochondrial apoptosis pathway. (**A**) The PARP and active caspase-3 protein levels in Jurkat cells were analyzed by Western blotting after 24 h of treatment. One representative Western blot is shown. (**B**,**C**) The Mcl-1 and Bax protein levels in Jurkat (**B**) and MCF-7 (**C**) cells were analyzed by Western blotting after 24 h of treatment. One representative Western blot is shown. (**D**,**E**) Jurkat (**D**) and MCF-7 (**E**) cells were treated as indicated for 24 h, followed by JC-1 staining. The loss of mitochondrial membrane potential was quantified by determining the percentage of green fluorescence caused by JC-1 monomers via flow cytometry. (**F**,**G**) Jurkat (**F**) and MCF-7 (**G**) cells were treated as indicated for 24 h, followed by MitoSOX^TM^ Red Superoxide Indicator staining. Mitochondrial superoxide was determined by flow cytometry analysis and displayed as the geometric mean of fluorescent intensity (gMFI). The bar graphs display the mean ± SD (*n* = 3). Circles indicate individual data points. One-way ANOVA was conducted, followed by Tukey’s multiple comparison test, referring to control. * *p* <0.05, ** *p* < 0.005, **** *p* < 0.0001 The graphics were in part taken (and modified) from the PhD thesis of F.L.

To verify the cell death induction capacity of zephycandidine A and analogues, we analyzed established markers for apoptosis. Firstly, we analyzed the activation of effector caspase-3 [17] and its downstream substrate, the DNA repair protein PARP (poly [ADP-ribose] polymerase) [18], using a Western blot analysis. While the lead structure zephycandidine A exhibits neither detectable caspase-3 activation nor significant PARP cleavage, the closely related compound **11** displays the activation of caspase-3, accompanied by PARP cleavage (Figure 3A), indicating significant apoptosis induction.

As our analogues induce apoptosis, we were interested in the mode of cell death induction. An early step in apoptosis is transcriptionally activating pro-apoptotic Bcl-2 family members (e.g., Bax, Bak, Bcl-xS, and Mcl-1S) and repressing anti-apoptotic Bcl-2 proteins (e.g., Mcl-1L, Bcl-2, and Bcl-xL). We checked for the expression of Bax and Mcl-1L protein levels after treatment with zephycandidine A and selected analogues. An analysis of the protein level in Jurkat cells (Figure 3B) revealed the downregulation of anti-apoptotic Mcl-1L and the upregulation of pro-apoptotic Bax upon treatment with compound **11**, whereas zephycandidine A did not display an effect. In breast cancer MCF7 cells, no effect of zephycandidine A or analogues on Bcl-2 family proteins was detected (Figure 3C).

Mechanistically, Mcl-1L and Bax interact with each other, as do other members of the Bcl-2 family, to form a mitochondrial apoptosis-induced channel (MAC) in the mitochondrial outer membrane, leading to the release of cytochrome c and other pro-apoptotic factors from the mitochondria. Due to the subsequent apoptosome formation and activation of caspase 9, effector caspases 3 and 7 are activated, thereby driving the apoptosis process [19,20]. Along the line, Zhan et al. [2] described that carrying out zephycandidine A treatment on HL-60 cells led to an upregulation of Bax as well as a downregulation of anti-apoptotic Bcl-2, already implying the involvement of mitochondria in the mediation of apoptosis. To assess whether mitochondria are affected by our compounds, we analyzed mitochondrial integrity by flow cytometry. Therefore, we investigated whether zephycandidine A and its analogues facilitate the loss of mitochondrial membrane potential (Δψm) and the formation of mitochondrial superoxides, an indicator for mitochondrial oxidative stress. Δψm can be detected by JC-1 staining. If Δψm is intact, JC-1 is capable of accumulating in the healthy mitochondria, forming aggregates that elicit a red fluorescence signal. In contrast, damaged mitochondria show mitochondrial depolarization indicated by a decreased red/green fluorescence intensity. We found a significant increase in dissipated mitochondria in both Jurkat and MCF-7 cells upon treatment with analogue **11**, whereas Δψm was unaffected by zephycandidine A (Figure 3D,E). Using MitoSOX^TM^ Red (Thermo Fisher Scientific, Eugene, OR, USA), we revealed that concomitant treatment with compound **11** results in an increased presence of mitochondrial superoxide in both cell lines (Figure 3F,G). These data indicate that the lead compound zephycandidine A does not significantly induce apoptosis in the tested cell lines, but the novel analogue **11** causes apoptosis in a mitochondria-dependent manner.

**Figure 4:**
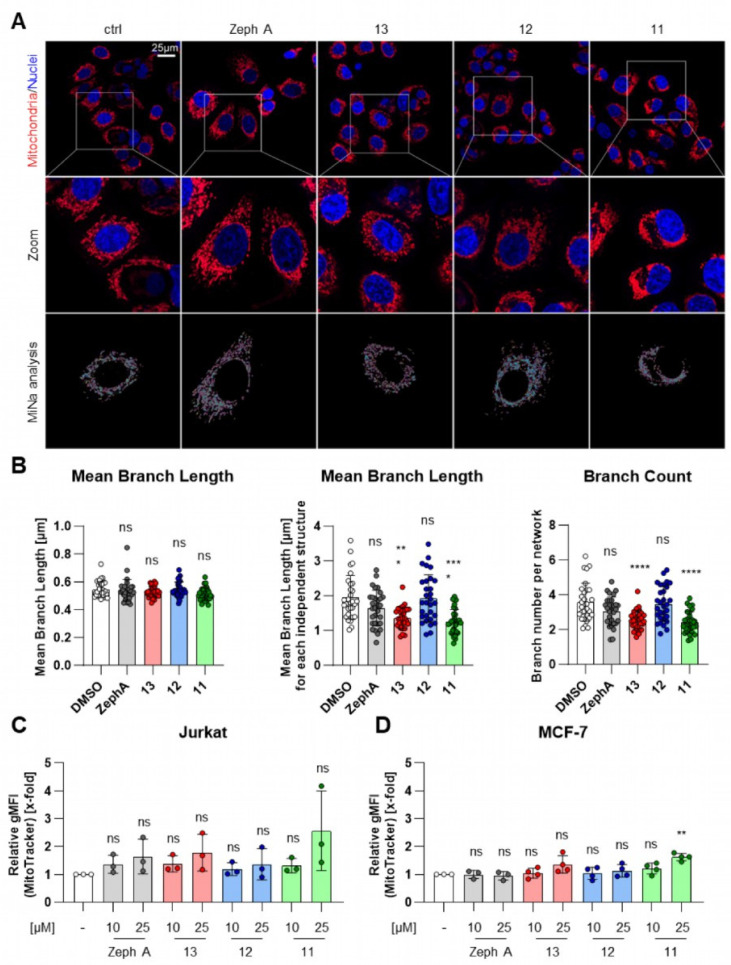
Zephycandidine A analogues alter mitochondrial morphology and increase mitochondrial mass. (**A**) MCF-7 cells were treated at indicated concentrations for 24 h before staining with MitoTracker^TM^ Red CMXRos (mitochondria) and Hoechst 33342 (nuclei). Mitochondrial morphology was assessed using confocal imaging. Scale bar: 25 µM. One representative image is shown. In addition, ImageJ plugin MiNA analysis output image is depicted. Purple area represents mitochondrial signal. Green lines represent length of mitochondria. Blue dots mark connection sites between mitochondria, and yellow dots mark end of network structures. (**B**) Results from MiNA analysis of (**A**) performed on 10 cells per replicate. (**C**,**D**) Jurkat (**C**) and MCF-7 (**D**) cells were treated as indicated for 24 h followed by MitoTracker^TM^ Green FM staining. Mitochondrial mass was determined by flow cytometry analysis and displayed as geometric mean of fluorescent intensity (gMFI). Bar graphs display mean ± SD (*n* = 3). Circles indicate individual data points. One-way ANOVA was conducted, followed by Tukey’s multiple comparison test, referring to control. * *p* < 0.05, ** *p* < 0.005, *** *p* < 0.001, **** *p* < 0.0001 Graphics were in part taken (and modified) from PhD thesis of F.L [27].

To confirm the effect of compound **11** on mitochondrial function, we further analyzed mitochondrial morphology and biogenesis. Several studies report that the mitochondrial morphology greatly affects mitochondrial function [21,22,23]; hence, we observed mitochondrial morphology by confocal imaging. Mitotracker staining revealed that the control cells displayed fibre-shaped, elongated mitochondria, which transformed to round-shaped structures upon treatment with compound **11** (Figure 4A,B). This phenotype was also observed in the literature upon mitochondria-driven apoptosis, where it was reported to be induced by a reduction in cristae density, mtDNA depletion, and a disbalance of mitochondrial dynamics that finally aggravate mitochondrial function [23,24,25]. As a response to mitochondrial dysfunction, cells frequently increase mitochondrial biogenesis [26]. Thus, we assessed the mitochondrial mass upon treatment, with no evident induction of mitochondrial biogenesis upon treatment with zephycandidine A or analogues (Figure 4C,D). In sum, compound **11** induces the intrinsic apoptosis pathway (Figure 3A–G and Figure 4A,B) without inducing a regulatory upregulation in mitochondrial mass (Figure 4C,D), leading to cell death in leukemia cells (Figure 2F–I).

### 2.3. Discussion 

In this study, we developed short approaches to obtain zephycandidine A (**3**) and related imidazo[1,2-*f*]phenanthridines with modifications of substituents on benzenoid ring A and imidazole ring D. We showed the antiproliferative properties of the parent alkaloid and novel synthetic analogues in four different cancer cell lines. We investigated the capacity of the parental molecule and its analogues to enable cell death and found that the novel analogue THK-121 (**11**) leads to the induction of mitochondrial apoptosis, while the parental alkaloid does not. We hence described novel zephycandidine A analogues with increased cytotoxic potential, preferentially targeting cancer cells. This study will enable a further analysis of zephycandidine A and a library of analogues on their biological activity, for instance, in cancer cell migration. Interestingly, in that regard, Narva et al. showed that synthetic analogues of zephycandidine A (**3**), mostly with additional substituents at ring D, not only facilitate antitumoral potential by inhibiting proliferation and inducing apoptosis but also by inhibiting cancer cell migration [10]. Since dysfunctional mitochondria have been linked excessively to metastasis and migration [28], it would be interesting to check zephycandidine A analogues on their potential to interfere with migration.

## 3. Experimental Procedure

### 3.1. Chemistry

#### General Reagent and Analytical Information

All solvents used were purified according to standard procedures or were of HPLC or p.a. grade and purchased from commercial sources. Chemical reagents were purchased from Sigma Aldrich (Schnelldorf, Germany), TCI (Eschborn, Germany), and BLD Pharm (Kaiserslautern, Germany). IR spectra were recorded on a Perkin Elmer (Waltham, MA, USA) FTIR Paragon 1000 spectrometer. NMR spectra were recorded on Jeol (Tokyo, Japan) JNMR-GX 400 (400 MHz), Jeol JNMR-GX 500 (500 MHz), Avance III HD Bruker BioSpin (400 MHz) (Brker, Billerica, MA, USA), and Avance III HD Bruker BioSpin (500 MHz) spectrometers. Spectra were recorded in deuterated solvents, and signal assignments were carried out based on ^1^H, ^13^C, DEPT, HMQC, HMBC, and COSY spectra. Chemical shifts are reported in parts per million (ppm), and J values are reported in hertz. High-resolution mass spectra were performed by electrospray ionization (ESI) using a Thermo Finnigan (San Josa, CA, USA) LTQ FT Ultra spectrometer or electron impact (EI) at 70 eV on a Jeol GCmate II or on a Finnigan MAT 95 spectrometer. All reactions were monitored by GC/MS or thin-layer chromatography (TLC) using POLYGRAM^®^SIL G/UV254 precoated plastic sheets from Macherey–Nagel (Düren, Germany). Compounds on TLC plates were detected under UV light at 254 and 366 nm. Separations with flash column chromatography (FCC) were performed on Merck silica gel 60 as stationary phase. Melting points were determined using open tube capillary method on a Büchi melting point B-540 apparatus and were uncorrected. HPLC purities were determined using an HP Agilent 1100 HPLC (Agilent, Waldbronn, Germany) with a diode array detector and an Agilent Zorbax Eclipse plus C18 column (150 × 4.6 mm; 5 μm) with acetonitrile/water in different proportions as mobile phase.

**General procedure A for conversion of aromatic aldehydes into nitriles.** The aldehyde **5a–e** (10 mmol, 1.0 eq) was dissolved in DMSO, and hydroxylamine hydrochloride (15 mmol, 1.5 eq) was added. The mixture was heated to 90 °C and stirred for 1 h at this temperature. After cooling to room temperature, water (20 mL) was added, and the suspension was extracted with ethyl acetate (3 × 15 mL). The organic phases were pooled, washed with brine (3 × 15 mL), dried over Na_2_SO_4_, and evaporated. The crude product was purified by silica gel column chromatography with the declared eluent.

**General procedure B for synthesis of aminophenanthridines 7a–7e.** Substituted 2-bromobenzonitrile **6a–e** (2.5 mmol and 1.0 eq), 2-aminophenylboronic acid pinacol ester (**7**) (548 mg, 2.50 mmol, and 1.00 eq), and bis(triphenylphosphine)palladium(II) chloride (88 mg, 0.013 mmol, and 0.050 eq) were dissolved in 15 mL of dry, degassed DMF in a Schlenk round-bottom flask under N_2_. Afterwards, 4 mL of a shortly degassed aqueous 2 M Na_2_CO_3_ solution was added, and the yellow suspension was stirred at 80 °C for 16 h. After cooling to room temperature, the crude mixture was filtrated through a short plug of Celite, which was subsequently washed with 50 mL methylene chloride and 50 mL methylene chloride/methanol at a ratio of 9:1. Then, the combined organic solution was mixed with 100 mL water, and the organic phase was separated. The aqueous phase was extracted twice with 25 mL methylene chloride, and the organic fractions were pooled, dried over anhydrous Na_2_SO_4_, and concentrated in vacuo. The crude product was purified by silica gel column chromatography with the declared eluent.

**General procedure C for cyclization to imidazophenanthridine ring system.** Substituted 6-aminophenanthridine **8a–e** (0.42 mmol, 1.0 eq), chloroacetaldehyde (50% in water, 0.11 mL, and 4.3 eq), and Na_2_CO_3_ (71 mg, 0.84 mmol, and 2.0 eq) were mixed with 3 mL isopropanol and 3 mL water. The suspension was refluxed for 1 h. After cooling to room temperature, 10 mL of water was added, and the suspension was extracted with ethyl acetate (3 × 10 mL). The organic phases were pooled, washed with brine (10 mL), dried over Na_2_SO_4_, and evaporated. The crude product was purified by silica column chromatography with the declared eluent.

**General procedure D for cyclization to ring D-substituted imidazophenanthridines 13–15.** Aminophenanthridine **8a** (48 mg, 0.20 mmol, 1.0 eq) and sulfur (103 mg, 3.20 mmol, and 16.0 eq) were suspended in 1 mL DMSO/cyclohexane (2:1). The appropriate amount of aldehyde (0.30 mmol, 1.5 eq) was added, and the mixture was heated at 120 °C overnight. After cooling to room temperature, water (10 mL) was added, and the black mixture was extracted with methylene chloride (3 × 10 mL). The combined organic phases were washed with brine (2 × 10 mL), dried over Na_2_SO_4_, and evaporated. The crude product was purified by silica column chromatography with the declared eluent.

*6-Bromobenzo[d][1,3]dioxole-5-carbonitrile* (**6a**). The synthesis was accomplished following general procedure A starting from aldehyde **5a**. The eluent for FSC isohexane/ethyl acetate at a 2:1 ratio was used to afford **6a** as a colourless solid (2.17 g, 9.05 mmol, 96%). m.p. 134–135 °C. ^1^H NMR (400 MHz, CDCl_3_) δ 7.09 (s, 1H, 6-H), 7.03 (s, 1H, 3-H), 6.10 (s, 2H, -OCH_2_O-). ^13^C NMR (101 MHz, CDCl_3_) δ 152.25 (C-2), 147.47 (C-1), 119.00 (C-4), 117.36 (-CN), 113.43 (C-6), 112.59 (C-3), 107.98 (C-5), 103.06 (-OCH_2_O-). IR (ATR): ṽ_max_/cm^−1^ = 2913, 2231, 1495, 1480, 1259, 1111, 1031, 922, 880, 838, 737. HRMS (EI): calcd. for C_8_H_4_BrNO_2_ (M)^•+^: 224.9425; found: 224.9423. 

*2-Bromo-4,5-dimethoxybenzonitrile* (**6b**). Synthesis was accomplished following general procedure A from 2-bromo-4,5-dimethoxybenzaldehyde (**5b**). The eluent for FSC isohexane/ethyl acetate at a ratio of 5:1 was used to afford **6b** as a colourless solid (2.23 g, 9.22 mmol, and 92%). m.p. 117–119 °C. ^1^H NMR (400 MHz, methylene chloride-*d*_2_) δ 7.10 (s, 1H, 3-H), 7.07 (s, 1H, 6-H), 3.89 (s, 3H, 1′-H), 3.84 (s, 3H, 2’H). ^13^C NMR (101 MHz, methylene chloride-*d*_2_) δ 153.46 (C-4), 148.74 (C-5), 117.62 (C-2), 117.21 (-CN), 115.53 (C-6), 115.44 (C-3), 106.63 (C-1), 56.41 (C-1’), 56.27 (C-2’). IR (ATR): ṽ_max_/cm^−1^ = 2942, 2229, 1592, 1505, 1377, 1261, 1219, 1168, 1035, 952, 874, 853, 794. HRMS (EI): calcd. for C_9_H_8_BrNO_2_ (M)^•+^: 240.9733; found: 240.9736. 

*2-Bromo-5-hydroxy-4-methoxybenzonitrile.* Synthesis was accomplished following general procedure A from commercially available 2-bromo-5-hydroxy-4-methoxybenzaldehyde. The eluent for FSC isohexane/ethyl acetate at a ratio of 1:1 was used to afford the target compound as a colourless solid (2.19 g, 9.62 mmol, and 96%). m.p. 160–163 °C. ^1^H NMR (400 MHz, DMSO-*d*_6_) δ 10.07 (s, 1H, -OH), 7.35 (s, 1H, 6-H), 7.17 (s, 1H, 3-H), 3.87 (s, 3H, -OCH_3_). ^13^C NMR (101 MHz, DMSO-*d*_6_) δ 152.69 (C-4), 146.49 (C-5), 119.39 (C-3), 117.70 (C-2), 116.24 (C-6), 114.44 (-CN), 105.35 (C-1), 56.40 (-OCH_3_). IR (ATR): ṽ_max_/cm^−1^ = 3367, 2229, 1608, 1508, 1438, 1286, 1267, 1211, 1160, 1020, 867, 842, 804. HRMS (EI): calcd. for C_8_H_6_BrNO_2_ (M)^•+^: 226.9576; found: 226.9582.

*2-Bromo-5-isopropoxy-4-methoxybenzonitrile* (**6d**). 2-Bromo-5-hydroxy-4-methoxybenzonitrile (1.14 g, 5.00 mmol, and 1.00 eq) was dissolved in dry acetone (50 mL), K_2_CO_3_ (1.24 g, 7.50 mmol, and 1.50 eq) was added, and the resulting suspension was stirred for 15 min. 2-Iodopropane (1.5 mL, 15 mmol, 3.0 eq) was added, and the mixture was stirred for 48 h at 50 °C. Brine (100 mL) was added, and the suspension extracted with ethyl acetate (3 × 50 mL). The organic phases were pooled, washed with brine (50 mL), dried over Na_2_SO_4_, and evaporated, resulting in a yellow oil, which was purified by silica column chromatography (isohexane/ethyl acetate at a ratio of 2:1) to afford **6d** as a colourless solid (1.28 g, 4.73 mmol, and 95%). m.p. 89–91 °C. ^1^H NMR (400 MHz, methylene chloride-*d*_2_) δ 7.10 (s, 1H, 3-H, or 6-H), 7.09 (s, 1H, 3-H, or 6-H), 4.49 (hept, *J* = 6.1 Hz, 1H, 1’-H), 3.87 (s, 3H, -OCH_3_), 1.33 (d, *J* = 6.1 Hz, 6H, 2’-H). ^13^C NMR (101 MHz, methylene chloride-*d*_2_) δ 154.68 (C-4), 146.83 (C-5), 119.18 (C-6), 117.65 (C-2), 117.10 (-CN), 116.01 (C-3), 106.62 (C-1), 72.16 (C-1’), 56.37 (-OCH_3_), 21.52 (C-2’). IR (ATR): ṽ_max_/cm^−1^ = 2922, 2223, 1588, 1504, 1437, 1377, 1268, 1259, 1217, 1166, 1138, 1027, 921, 860, 798. HRMS (EI): calcd. for C_9_H_8_BrNO_2_ (M)^•+^: 269.0046; found: 269.0049.

*2-Bromonicotinonitrile* (**6e**). Synthesis was accomplished following general procedure A from commercially available 2-bromo-3-pyridinecarboxaldehyde. The eluent for FSC isohexane/ethyl acetate at a ratio of 5:1 was used to afford **6e** as a colourless solid (1.72 g, 9.41 mmol, and 94%). m.p. 107–109 °C. ^1^H NMR (400 MHz, methylene chloride-*d*_2_) δ 8.60 (dd, *J* = 4.9, 1.9 Hz, 1H, 6-H), 8.03 (dd, *J* = 7.7, 2.0 Hz, 1H, 4-H), 7.41 (dd, *J* = 7.7, 4.9 Hz, 1H, 5-H). ^13^C NMR (101 MHz, methylene chloride-*d*_2_) δ 152.92 (C-6), 152.52 (C-2), 142.72 (C-4), 122.38 (C-5), 114.67 (-CN), 110.82 (C-3). IR (ATR): ṽ_max_/cm^−1^ = 3081, 3065, 2236, 1577, 1398, 1145, 1131, 1079, 807, 7356, 672. HRMS (EI): calcd. for C_6_H_3_BrN_2_ (M)^•+^: 181.9474; found: 181.9478.

*[1,3]Dioxolo[4,5-j]phenanthridin-6-amine* (**8a**). Synthesis was accomplished following general procedure B starting from nitrile **6a**. The eluent for FSC isohexane/ethyl acetate at a ratio of 1:2 (containing 1% triethylamine) was used to afford **8a** as a beige solid (455 mg, 1.91 mmol, and 76%). m.p. 250–252 °C. ^1^H NMR (400 MHz, DMSO-*d*_6_) δ 8.35 (dd, *J* = 8.2, 1.4 Hz, 1H, 4-H), 8.13 (s, 1H, 11-H), 7.81 (s, 1H, 7-H), 7.52–7.39 (m, 2H, 1-H, 3-H), 7.21 (ddd, *J* = 8.3, 6.8, 1.5 Hz, 1H, 2-H), 6.78 (s, 2H, NH_2_), 6.22 (s, 2H, OCH_2_O). ^13^C NMR (101 MHz, DMSO-d_6_), ^13^C NMR (101 MHz, DMSO-*d*_6_) δ 155.06 (C-6), 150.41 (C-7a or C-10a), 147.63 (C-7a or C-10a), 144.56 (C-4a), 130.71 (C-11a), 127.81 (C-3), 125.50 (C-1), 122.37 (C-4), 121.44 (C-2), 120.58 (C-11b), 114.07 (C-6a), 102.25 (C-7), 101.91 (OCH_2_O), 100.81 (C-11). IR (ATR): ṽ_max_/cm^−1^ = 3484, 3059, 1660, 1454, 1233, 1035, 1029, 938, 753, 731. HRMS (EI): calcd. for C_14_H_10_N_2_O_2_ (M)^•+^: 238.0737; found: 238.0738.

*8,9-Dimethoxyphenanthridin-6-amine* (**8b**). Synthesis was accomplished following general procedure B starting from nitrile **6b**. The eluent for FSC ethyl acetate/triethylamine at a ratio of 99:1 was used to afford **8b** as an off-white solid (403 mg, 1.58 mmol, and 63%). m.p. 215–216 °C. ^1^H NMR (500 MHz, methylene chloride-*d*_2_) δ 8.29 (dd, *J* = 8.1, 1.4 Hz, 1H, 4-H), 7.86 (s, 1H, 7-H), 7.66 (dd, *J* = 8.2, 1.4 Hz, 1H, 1-H), 7.52 (ddd, *J* = 8.2, 7.0, 1.4 Hz, 1H, 2-H), 7.37 (ddd, *J* = 8.2, 7.0, 1.4 Hz, 1H, 3-H), 7.20 (s, 1H, 10-H), 5.20–5.15 (br s, 2H, -NH_2_), 4.08 (s, 3H, 2’-H), 4.00 (s, 3H, 1’-H). ^13^C NMR (126 MHz, methylene chloride-*d*_2_) δ 154.57 (C-6), 153.15 (C-9), 150.29 (C-8), 144.57 (C-4a), 130.00 (C-10a), 128.47 (C-3), 127.20 (C-2), 123.30 (C-1), 122.07 (C-4), 121.89 (C-10b), 113.56 (C-6a), 104.27 (C-10), 103.57 (C-7), 56.59 (C-2’), 56.51 (C-1’). IR (ATR): ṽ_max_/cm^−1^ = 3384, 3155, 1661, 1615, 1524, 1451, 1359, 1266, 1206, 1021, 803, 749, 732. HRMS (EI): calcd. for C_15_H_14_N_2_O_2_ (M)^•+^: 254.1050; found: 254.1051.

*8-Methoxyphenanthridin-6-amine* (**8c**). Synthesis was accomplished following general procedure B starting from commercially available 2-bromo-5-methoxybenzonitrile (**6c**). The eluent for FSC ethyl acetate/triethylamine at a ratio of 99:1 was used to afford **8c** as an off-white solid (450 mg, 2.01 mmol, and 80%). m.p. 174–175 °C. ^1^H NMR (400 MHz, DMSO-*d*_6_) δ 8.56 (d, *J* = 9.0 Hz, 1H, 10-H), 8.37 (dd, *J* = 8.2, 1.4 Hz, 1H, 4-H), 7.77 (d, *J* = 2.6 Hz, 1H, 7-H), 7.51 (dd, *J* = 8.2, 1.4 Hz, 1H, 1-H), 7.43 (m, 2H, 9-H, 2-H), 7.24 (ddd, *J* = 8.2, 6.8, 1.4 Hz, 1H, 3-H), 6.99 (s, 2H, NH_2_), 3.93 (s, 3H, OCH_3_). ^13^C NMR (101 MHz, DMSO-*d*_6_) δ 158.45 (C-8), 155.11 (C-6), 143.97 (C-4a), 127.52 (C-10a), 127.31 (C-3), 125.46 (C-2), 124.28 (C-4), 121.73 (C-9, C-10), 120.37 (C-10b), 120.19 (C-1), 119.89 (C-6a), 105.82 (C-7), 55.70 (-OCH_3_). IR (ATR): ṽ_max_/cm^−1^ = 3392, 3099, 1616, 1532, 1407, 1345, 1254, 1221, 1035, 999, 821, 752, 745, 729. HRMS (EI): calcd. for C_14_H_12_N_2_O (M)^•+^: 224.0944; found: 224.0950.

*8-Isopropoxy-9-methoxyphenanthridin-6-amine* (**8d**). Synthesis was accomplished following general procedure B starting from nitrile **6d**. The eluent for FSC ethyl acetate/triethylamine at a ratio of 99:1 was used to afford **8d** as an off-white solid (564 mg, 2.00 mmol, and 80%). m.p. 208–210 °C. ^1^H NMR (400 MHz, DMSO-*d*_6_) δ 8.42 (dd, *J* = 8.2, 1.4 Hz, 1H, 4-H), 7.97 (s, 1H, 7-H), 7.74 (s, 1H, 10-H), 7.51–7.37 (m, 2H, 1-H, 3-H), 7.22 (ddd, *J* = 8.2, 6.8, 1.5 Hz, 1H, 2-H), 6.85 (s, 2H, -NH_2_), 4.86 (hept, *J* = 6.1 Hz, 1H, 1’-H), 4.01 (s, 3H, -OCH_3_), 1.34 (d, *J* = 6.0 Hz, 6H, 2’-H). ^13^C NMR (101 MHz, DMSO-*d*_6_) δ 155.42 (C-6), 153.19 (C-9), 147.54 (C-8), 144.97 (C-4a), 129.05 (C-10a), 127.96 (C-3), 125.91 (C-1), 122.69 (C-4), 121.65 (C-2), 120.79 (C-10b), 113.40 (C-6a), 108.52 (C-10), 104.04 (C-7), 70.66 (C-1’), 56.25 (-OCH_3_), 22.32 (C-2’). IR (ATR): ṽ_max_/cm^−1^ = 3460, 3133, 1646, 1506, 1450, 1263, 1203, 1106, 1022, 850, 761, 737 HRMS (EI): calcd. for C_17_H_18_N_2_O_2_ (M)^•+^: 282.1368; found: 282.1358.

*Benzo[h][1,6]naphthyridin-5-amine* (**8e**). Synthesis was accomplished following general procedure B starting from nitrile **6e**. The eluent for FSC ethyl acetate/triethylamine at a ratio of 99:1 was used to afford **8e** as an off-white solid (475 mg, 2.43 mmol, and 97%). m.p. 128–130 °C. ^1^H NMR (400 MHz, DMSO-*d*_6_) δ 9.09 (dd, *J* = 4.4, 1.6 Hz, 1H, 2-H), 8.75 (m, 2H, 4-H, 10-H), 7.71 (ddd, *J* = 8.3, 4.4, 0.7 Hz, 1H, 3-H), 7.64–7.53 (m, 2H, 7-H, 8-H), 7.32 (ddd, *J* = 8.2, 6.8, 1.4 Hz, 1H, 9-H), 7.23 (s, 2H, -NH_2_). ^13^C NMR (101 MHz, DMSO-*d*_6_) δ 156.05 (C-5), 153.16 (C-2), 149.71 (C-10b), 147.48 (C-6a), 133.25 (C-4), 130.72 (C-8), 125.72 (C-7), 123.71 (C-10), 122.85 (C-3), 122.40 (C-9), 121.77 (C-10a), 114.25 (C-4a). IR (ATR): ṽ_max_/cm^−1^ = 3102, 1652, 1470, 1104, 740. HRMS (EI): calcd. for C_12_H_9_N_3_ (M)^•+^: 195.0791; found: 195.0791.

*[1,3]Dioxolo[4,5-j]imidazo[1,2-f]phenanthridine (***3***, zephycandidine A).* Synthesis was accomplished following general procedure C using **8a** as substituted phenanthridine. The eluent for FSC isohexane/ethyl acetate at a ratio of 1:1 was used to afford **3** as a colourless solid (98 mg, 0.37 mmol, and 89%). m.p. 242–244 °C (lit. [4]: 242–243 °C, lit. [5]: 242–245 °C). ^1^H NMR (400 MHz, CDCl_3_) δ 8.24 (dd, J = 8.1, 1.4 Hz, 1H, 8-H), 8.02 (s, 1H, 9-H), 7.95 (d, J = 1.5 Hz, 1H, 2-H), 7.84 (dd, J = 8.2, 1.3 Hz, 1H, 5-H), 7.71 (s, 1H, 13-H), 7.60–7.54 (m, 2H, 3-H, 6-H), 7.48 (ddd, J = 8.4, 7.2, 1.3 Hz, 1H, 7-H), 6.13 (s, 2H, -OCH_2_O-). ^13^C NMR (101 MHz, CDCl_3_) δ 149.42 (C-9a), 148.76 (C-12), 142.63 (C-13b), 131.34 (C-2), 131.11 (C-4a), 128.01 (C-6), 124.96 (C-7), 123.83 (C-8), 123.31 (C-8b), 121.75 (C-13a), 119.50 (C-8a), 115.84 (C-5), 111.53 (C-3), 102.88 (C-9), 101.76 (-OCH_2_O-), 101.39 (C-13). IR (ATR): ṽ_max_/cm^−1^ = 2920, 1506, 1462, 1331, 1260, 1035, 848, 736 HRMS (EI): calcd. for C_16_H_10_N_2_O_2_ (M)^•+^: 262.0737; found: 262.0737. HPLC purity: >99%.

*10,11-Dimethoxyimidazo[1,2-f]phenanthridine* (**9**). Synthesis was accomplished following general procedure C using **8b** as substituted phenanthridine. The eluent for FSC ethyl acetate/triethylamine at a ratio of 99:1 was used to afford **9** as an off-white solid (99 mg, 0.36 mmol, and 85%). m.p. 165–166 °C. ^1^H NMR (400 MHz, methylene chloride-*d*_2_) δ 8.34 (dd, *J* = 8.2, 1.4 Hz, 1H, 5-H), 8.01–7.98 (m, 2H, 3-H, 12-H), 7.88 (dd, *J* = 8.2, 1.3 Hz, 1H, 8-H), 7.71 (s, 1H, 2-H), 7.59 (ddd, *J* = 8.3, 7.2, 1.4 Hz, 1H, 6-H), 7.51 (s, 1H, 9-H), 7.51 (td, *J* = 7.0, 1.3 Hz, 1H, 7-H), 4.05 (s, 3H, OCH_3_), 4.04 (s, 3H, OCH_3_). ^13^C NMR (101 MHz, methylene chloride-*d*_2_) δ 151.22 (C-10 or C-11), 151.20 (C-10 or C-11), 142.94 (C-12b), 131.84 (C-4a), 131.59 (C-2), 128.36 (C-6), 125.36 (C-8), 124.15 (C-7), 122.12 (C-8b), 121.99 (C-8a), 118.61 (C-12a), 116.48 (C-5), 112.23 (C-3), 105.40 (C-12), 104.50 (C-9), 56.64 (-OCH_3_), 56.53 (-OCH_3_). IR (ATR): ṽ_max_/cm^−1^ = 2830, 1615, 1516, 1478, 1455, 1274, 1212, 1161, 1142, 1033, 1017, 854, 793, 753, 711. HRMS (EI): calcd. for C_17_H_14_N_2_O_2_ (M)^•+^: 278.1055; found: 278.1050. HPLC purity: >99%.

*11-Methoxyimidazo[1,2-f]phenanthridine* (**10**). Synthesis was accomplished following general procedure C using **8c** as substituted phenanthridine. The eluent for FSC ethyl acetate/triethylamine at a ratio of 99:1 was used to afford **10** as an off-white solid (85 mg, 0.34 mmol, and 82%). m.p. 99 °C. ^1^H NMR (400 MHz, methylene chloride-*d*_2_) δ 8.40 (dd, *J* = 8.1, 1.4 Hz, 1H, 9-H), 8.32 (d, *J* = 9.0 Hz, 1H, 5-H), 8.06 (d, *J* = 2.8 Hz, 1H, 2-H), 8.04 (d, *J* = 1.4 Hz, 1H, 3-H), 7.90 (dd, *J* = 8.2, 1.3 Hz, 1H, 8-H), 7.60 (ddd, *J* = 8.4, 7.2, 1.5 Hz, 1H, 6-H), 7.56 (d, *J* = 1.3 Hz, 1H, 12-H), 7.52 (ddd, *J* = 8.4, 7.2, 1.3 Hz, 1H, 7-H), 7.25 (dd, *J* = 9.0, 2.8 Hz, 1H, 10-H), 4.00 (s, 3H, -OCH_3_). ^13^C NMR (101 MHz, methylene chloride-*d*_2_) δ 160.02 (C-11), 142.03 (C-12b), 130.75 (C-4a or C-2), 130.70 (C-4a or C-2), 127.83 (C-7), 125.26 (C-6), 124.73 (C-12a), 124.26 (C-9), 123.62 (C-5), 121.86 (C-8a), 121.09 (C-8b), 118.38 (C-10), 115.83 (C-8), 112.35 (C-3), 105.19 (C-12), 55.76 (-OCH_3_). IR (ATR): ṽ_max_/cm^−1^ = 3434, 1617, 1472, 1325, 1291, 1036, 862, 750. HRMS (EI): calcd. for C_16_H_12_N_2_O (M)^•+^: 248.0944; found: 248.0943. HPLC purity: 99%.

*11-Isopropoxy-10-methoxyimidazo[1,2-f]phenanthridine (***11***, THK-121).* Synthesis was accomplished following general procedure C using **8d** as substituted phenanthridine. The eluent for FSC ethyl acetate/triethylamine at a ratio of 99:1 was used to afford **11** as an off-white solid (107 mg, 0.35 mmol, and 83%). m.p. 73 °C. ^1^H NMR (400 MHz, methylene chloride-*d*_2_) δ 8.36 (dd, *J* = 8.1, 1.5 Hz, 1H, 5-H), 8.03 (s, 1H, 12-H), 8.00 (d, *J* = 1.5 Hz, 1H, 3-H), 7.90 (dd, *J* = 8.2, 1.3 Hz, 1H, 8-H), 7.76 (s, 1H, 9-H), 7.60 (ddd, *J* = 8.3, 7.2, 1.4 Hz, 1H, 7-H), 7.54–7.50 (m, 2H, 2-H, 6-H), 4.87 (hept, 1H, 1’-H), 4.05 (s, 3H, -OCH_3_), 1.45 (d, *J* = 6.1 Hz, 6H, 2’-H). ^13^C NMR (101 MHz, methylene chloride-*d*_2_) δ 152.09 (C-10), 149.45 (C-11), 142.96 (C-12b), 131.83 (C-4a), 131.50 (C-2), 128.33 (C-6), 125.40 (C-8), 124.15 (C-7), 122.20 (C-8b), 121.87 (C-8a), 118.62 (C-12a), 116.50 (C-5), 112.23 (C-3), 108.01 (C-12), 105.03 (C-9), 71.56 (C-1’), 56.60 (-OCH_3_), 22.30 (C-2’). IR (ATR): ṽ_max_/cm^−1^ = 2976, 1614, 1514, 1453, 1384, 1263, 1209, 1107, 1022, 953, 924, 849, 798, 718. HRMS (EI): calcd. for C_19_H_18_N_2_O_2_ (M)^•+^: 306.1363; found: 306.1364. HPLC purity: >99%.

*Benzo[h]imidazo[2,1-f][1,6]naphthyridine* (**12**). Synthesis was accomplished following general procedure C using **8e** as substituted phenanthridine. The eluent for FSC ethyl acetate/triethylamine at a ratio of 99:1 was used to afford **12** as an off-white solid (57 mg, 0.26 mmol, and 62%). m.p. 253–255 °C. ^1^H NMR (400 MHz, methylene chloride-*d*_2_) δ 9.06 (dd, *J* = 8.1, 1.5 Hz, 1H, 5-H), 8.91 (dd, *J* = 4.5, 1.8 Hz, 1H, 10-H), 8.85 (dd, *J* = 8.0, 1.8 Hz, 1H, 12-H), 8.07 (d, *J* = 1.4 Hz, 1H, 3-H), 7.91 (dd, *J* = 8.3, 1.1 Hz, 1H, 8-H), 7.74 (ddd, *J* = 8.4, 7.1, 1.5 Hz, 1H, 6-H), 7.63–7.54 (m, 3H, 2-H, 7-H, 11-H). ^13^C NMR (101 MHz, methylene chloride-*d*_2_) δ 150.09 (C-10), 144.68 (C-8b), 141.38 (C-12b), 133.19 (C-4a), 131.93 (C-2), 131.37 (C-12), 130.48 (C-6), 126.07 (C-8), 125.32 (C-7), 123.35 (C-11), 122.73 (C-8a), 119.42 (C-12a), 115.30 (C-5), 112.61 (C-3). IR (ATR): ṽ_max_/cm^−1^ = 3319, 3148, 1653, 1581, 1479, 1472, 1395, 1295, 1080, 750, 726. HRMS (EI): calcd. for C_14_H_9_N_3_ (M)^•+^: 219.0791; found: 219.0790. HPLC purity: 98%.

*3-Methyl-[1,3]dioxolo[4,5-j]imidazo[1,2-f]phenanthridine* (**13**). Synthesis was accomplished following general procedure D using propanal (23 µL and 0.30 mmol) as aldehyde. The eluent for FSC isohexane/ethyl acetate at a ratio of 1:1 was used to afford **13** as a colourless solid (29 mg, 0.11 mmol, and 53%). m.p. 180–182 °C. ^1^H NMR (400 MHz, methylene chloride-*d*_2_) δ 8.34 (dd, *J* = 8.5, 1.3 Hz, 1H, 5-H), 8.30 (dd, *J* = 8.1, 1.6 Hz, 1H, 8-H), 7.97 (s, 1H, 13-H), 7.73 (s, 1H, 9-H), 7.56 (ddd, *J* = 8.5, 7.2, 1.6 Hz, 1H, 7-H or 6-H), 7.49 (ddd, *J* = 8.3, 7.2, 1.3 Hz, 1H, 7-H or 6-H), 7.21 (q, *J* = 1.1 Hz, 1H, 2-H), 6.12 (s, 2H, -OCH_2_O-), 2.91 (d, *J* = 1.1 Hz, 3H, -CH_3_). ^13^C NMR (101 MHz, methylene chloride-*d*_2_) δ 149.78 (C-9a or C-12a), 149.34 (C-9a or C-12a), 143.79 (C-13b), 134.26 (C-8a), 131.40 (C-2), 127.96 (C-6), 125.67 (C-3), 124.96 (C-7), 124.30 (C-8), 123.39 (C-8a), 122.94 (C-4a), 120.64 (C-13a), 116.98 (C-5), 102.94 (C-13), 102.46 (-OCH_2_O-), 101.60 (C-9), 15.64 (-CH_3_). IR (ATR): ṽ_max_/cm^−1^ = 2874, 1504, 1445, 1380, 1291, 1247, 1040, 871, 744. HRMS (EI): calcd. for C_17_H_12_N_2_O_2_ (M)^•+^: 276.0893; found: 276.0898. HPLC purity: 96%.

*3-Isopropyl-[1,3]dioxolo[4,5-j]imidazo[1,2-f]phenanthridine* (**14**). Synthesis was accomplished following general procedure D using 3-methylbutanal (32 µL and 0.30 mmol) as aldehyde. The eluent for FSC isohexane/ethyl acetate at a ratio of 1:1 was used to afford **14** as a colourless solid (35 mg, 0.12 mmol, and 58%). m.p. 170–171 °C. ^1^H NMR (400 MHz, methylene chloride-*d*_2_) δ 8.28 (ddd, *J* = 10.9, 8.3, 1.1 Hz, 2H, 5-H, 8-H), 7.99 (s, 1H, 13-H), 7.72 (s, 1H, 9-H), 7.57 (ddd, *J* = 8.5, 7.1, 1.6 Hz, 1H, 7-H), 7.48 (ddd, *J* = 8.1, 7.1, 1.2 Hz, 1H, 6-H), 7.31 (d, *J* = 1.0 Hz, 1H, 2-H), 6.11 (s, 2H, -OCH_2_O-), 3.79 (hept, *J* = 6.7 Hz, 1H, 1’-H), 1.49 (d, *J* = 6.6 Hz, 6H, 2’-H). ^13^C NMR (101 MHz, methylene chloride-*d*_2_) δ 149.24 (C-12a), 148.74 (C-9a), 143.53 (C-13b), 136.84 (C-3), 133.46 (C-4a), 127.79 (C-2), 127.40 (C-7), 124.34 (C-6), 123.78 (C-8), 122.89 (C-8b), 122.60 (C-13a), 120.17 (C-8a), 117.12 (C-5), 102.47 (C-13), 101.88 (-OCH_2_O-), 100.92 (C-9), 27.56 (C-1’), 22.48 (C-2’). IR (ATR): ṽ_max_/cm^−1^ = 1459, 1247, 1041, 871, 744 HRMS (EI): calcd. for C_19_H_16_N_2_O_2_ (M)^•+^: 304.1206; found: 304.1203. HPLC purity: 96%.

*3-Phenyl-[1,3]dioxolo[4,5-j]imidazo[1,2-f]phenanthridine* (**15**). Synthesis was accomplished following general procedure D using phenylacetaldehyde (35 µL and 0.30 mmol) as aldehyde. The eluent for FSC isohexane/ethyl acetate at a ratio of 1:1 was used to afford **15** as a colourless solid (14 mg, 0.04 mmol, and 21%). m.p. 232–233 °C. ^1^H NMR (400 MHz, methylene chloride-*d*_2_) δ 8.27 (dd, *J* = 8.2, 1.5 Hz, 1H, 5-H), 8.04 (s, 1H, 13-H), 7.77 (s, 1H, 9-H), 7.57 (dd, *J* = 8.5, 1.2 Hz, 1H, 8-H), 7.55–7.49 (m, 5H, 2’-h, 3’-H, 4’-H, 5’-H, 6’-H), 7.40 (ddd, *J* = 8.3, 7.1, 1.2 Hz, 1H, 7-H), 7.38 (s, 1H, 2-H), 7.23–7.18 (m, 1H, 6-H), 6.15 (s, 2H, -OCH_2_O-). ^13^C NMR (101 MHz, methylene chloride-*d*_2_) δ 149.50 (C-12a), 148.83 (C-9a), 143.74 (C-13b), 132.50 (C-3), 132.42 (C-2), 132.28 (C-1’), 129.87 (C-3’, C-5’), 129.46 (C-4’), 128.76 (C-2’, C-6’), 128.48 (C-4a), 126.89 (C-6), 124.60 (C-7), 123.76 (C-5), 123.47 (C-8a), 122.51 (C-13a), 119.87 (C-8b), 117.85 (C-8), 102.63 (C-13), 101.99 (-OCH_2_O-), 101.16 (C-9). IR (ATR): ṽ_max_/cm^−1^ = 2904, 1449, 1237, 1036, 910, 860, 767, 754, 748, 704. HRMS (EI): calcd. for C_22_H_14_N_2_O_2_ (M)^•+^: 338.1050; found: 338.1051. HPLC purity: 95%.

### 3.2. Biological Investigations

#### 3.2.1. Cell Lines and Culture

CCRF-CEM cells were obtained from Prof. Maria Kavallaris [29] (University of New South Wales, Sydney, Australia); Jurkat, HL-60, and MCF7 were obtained from ATCC and were cultured in RPMI 1640 (PAN Biotech, Aidenbach, Germany) containing 10% FCS (PAN Biotech). Human umbilical vein endothelial cells (HUVECs) were purchased from Promocell (Heidelberg, Germany) and cultivated with ECGM Kit enhanced (PELO Biotech, Planegg, Germany) supplemented with 10% (FCS) (PAA Laboratories, Cölbe, Germany) and 1% penicillin/streptomycin/amphotericin B (all purchased from PAN Biotech). All cells were cultured at 37 °C with 5% CO_2_ with constant humidity. Cell line STR profiling was performed. None of the cell lines used are listed in the database of commonly misidentified cell lines maintained by ICLAC. All cells are proven to be mycoplasma-free quarterly.

#### 3.2.2. Functional Analysis of Biological Activity

All tested compounds were diluted in DMSO (Sigma Aldrich) to 10 mM stock solutions. In biological assays, the final DMSO concentration did not exceed 0.1%. Cell proliferation was assessed using the CellTiter-Blue cell viability assay as described previously [30]. Apoptosis was assessed by flow cytometry according to Nicoletti et al., and an analysis of the protein level was carried out by immunoblotting as described previously [30,31]. The mitochondrial membrane potential was assessed by flow cytometry, and morphology was assessed by confocal microscopy as described previously [30].

#### 3.2.3. Statistical Analysis

Experiments were carried out at least three times independently unless stated otherwise. Data represent mean ± standard deviation (SD) unless stated otherwise. Statistical significance was assessed by ordinary one-way ANOVA with Dunnet’s post-test using GraphPad Prism 8. Significance of differences in dose–response curves was analyzed using the comparison of fits function of GraphPad Prism 8. Results were considered significant for *p* < 0.05.

## 4. Conclusions

In summary, we developed short approaches to zephycandidine A (**3**) and related imidazo[1,2-*f*]phenanthridines with modifications of substituents on benzenoid ring A and imidazole ring D. We investigated the antiproliferative properties of the alkaloid and its synthetic analogues in four cancer cell lines and identified novel zephycandidine A analogues with increased cytotoxic potential. Hereby, we found that the novel analogue THK-121 (**11**) leads to the induction of mitochondrial apoptosis, while the parental alkaloid does not. This represents a first systematic analysis of structure-activities of zephycandidine A and closely related analogues.

## Data Availability

The experimental data, spectra, and protocols are stored in an electronic lab journal by the authors.

## Supplementary Materials

The following supporting information can be downloaded at https://www.mdpi.com/article/10.3390/molecules30030752/s1: ^1^H and ^13^C NMR spectra of compounds **6a/b/d/e, 8a–e, 3,** and **9–15**; Table S1: list of IC_50_ values.
