# Supplementary material for: Zephycandidine A and Synthetic Analogues—Synthesis and Evaluation of Biological Activity
Source: Molecules. 2025 Feb 6;30(3):752. doi: 10.3390/molecules30030752 (PMC11820727; doi:10.3390/molecules30030752)
Supplement: Supplementary file 1 [file molecules-30-00752-s001.zip › molecules-3447777-supplementary.pdf]

SUPPORTING INFORMATION

Zephycandidine A and Synthetic Analogues – Synthesis and Evaluation of Biological Activity

Thomas Klaßmüller, Florian Lengauer, Julia Blenninger, Franz Geisslinger, Karin Bartel*‡ and Franz Bracher*‡

Department of Pharmacy – Center for Drug Research, Ludwig-Maximilians University, Butenandtstr. 5-13, 81377 Munich, Germany

Content:

NMR data of the compounds 6a/b/d/e, 8a-e, 3, 9-15

Table S1: List of IC_{50} values

^{1}H NMR spectrum of compound 6a (400 MHz, $CDCl_3$)

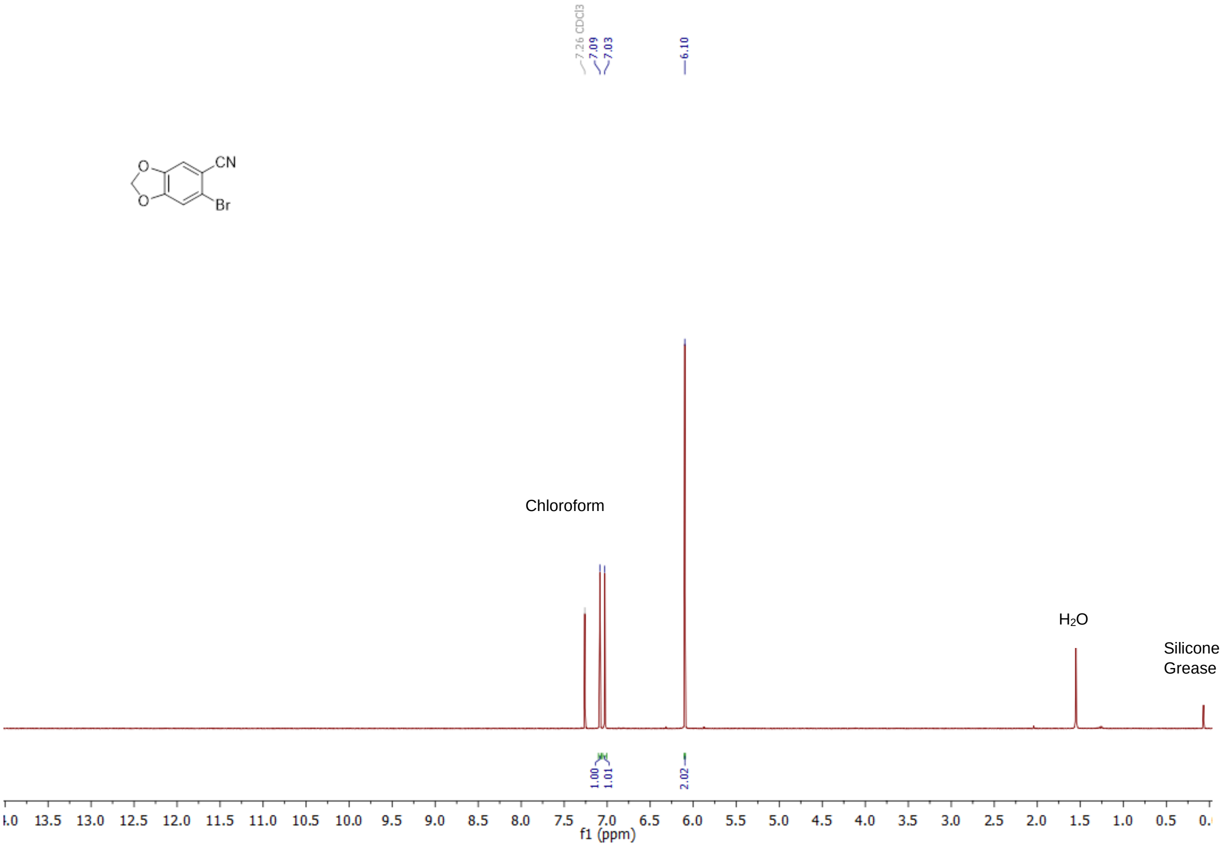

^{13}C NMR spectrum of compound 6a (101 MHz, $CDCl_3$)

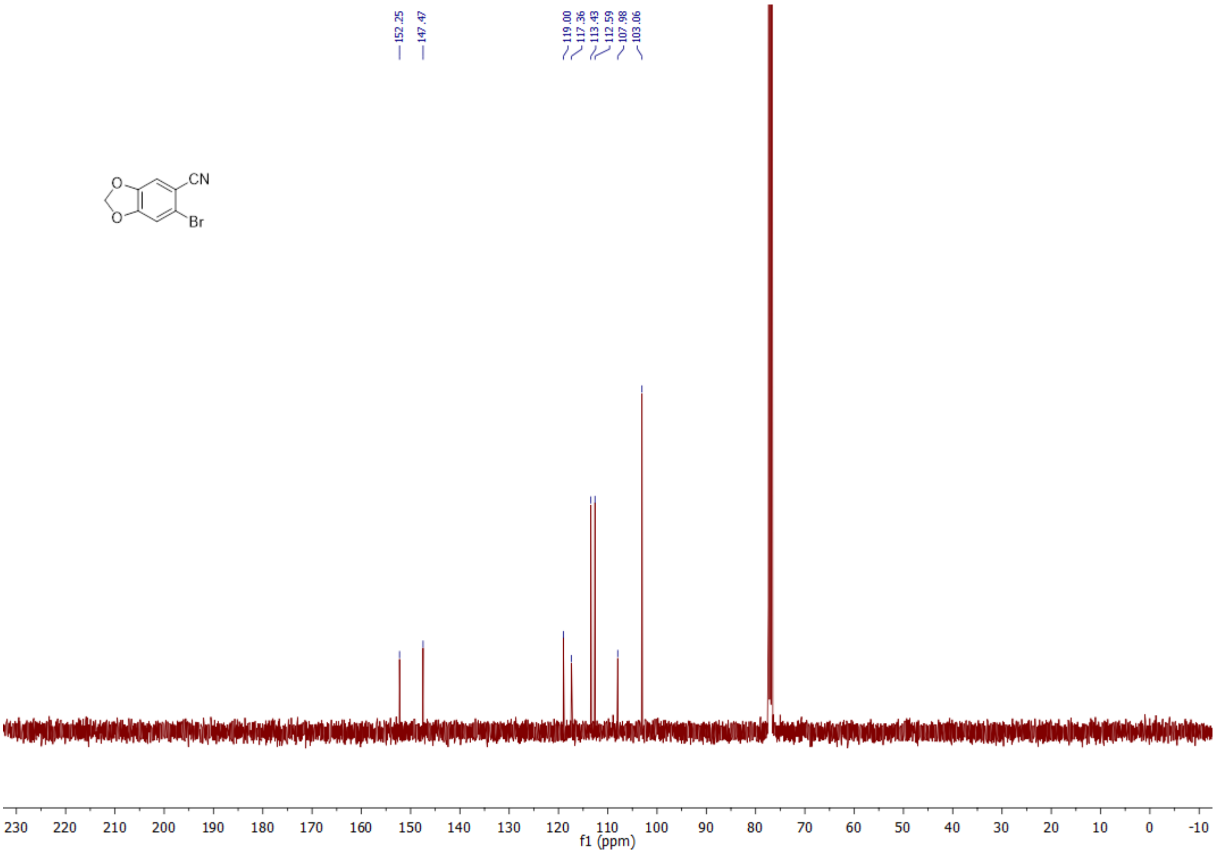

^{1}H NMR spectrum of compound 6b (400 MHz, methylene chloride-d_2)

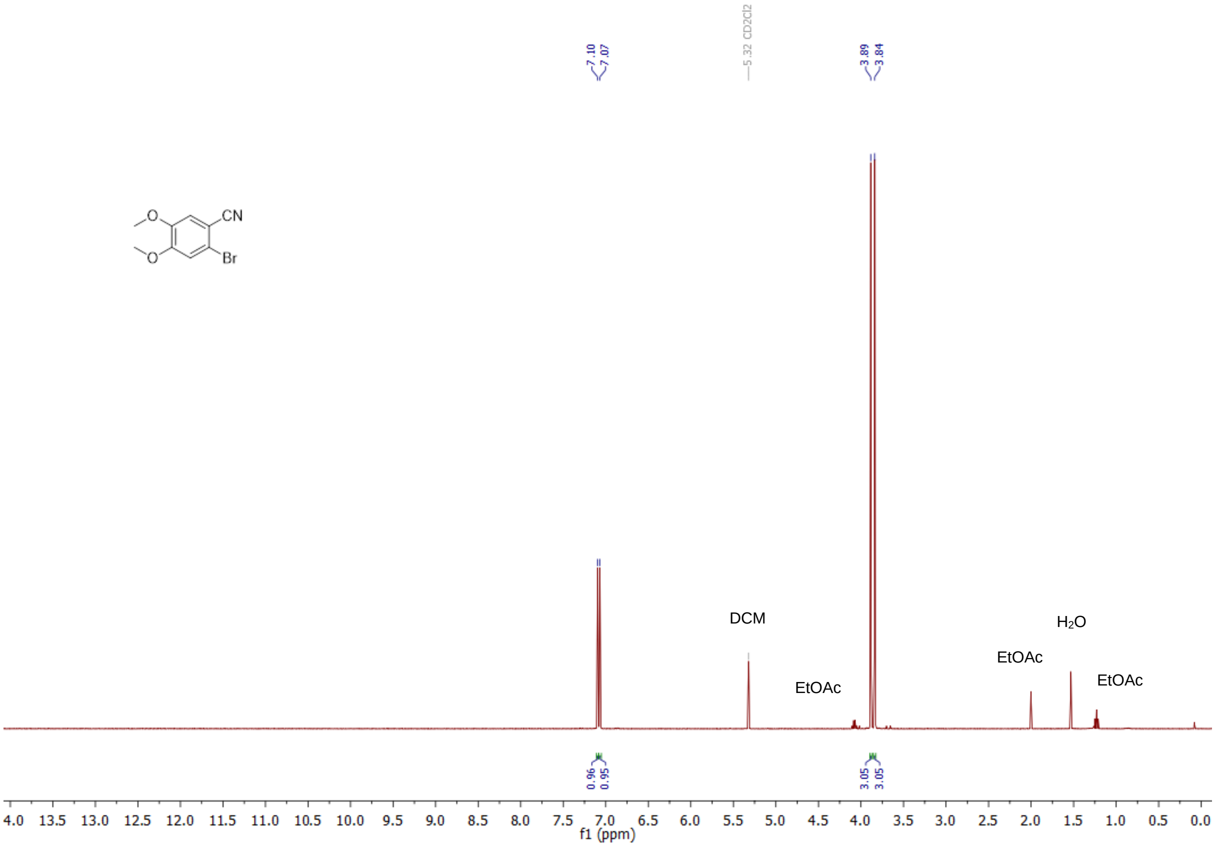

^{13}C NMR spectrum of compound 6b (101 MHz, methylene chloride-d_2)

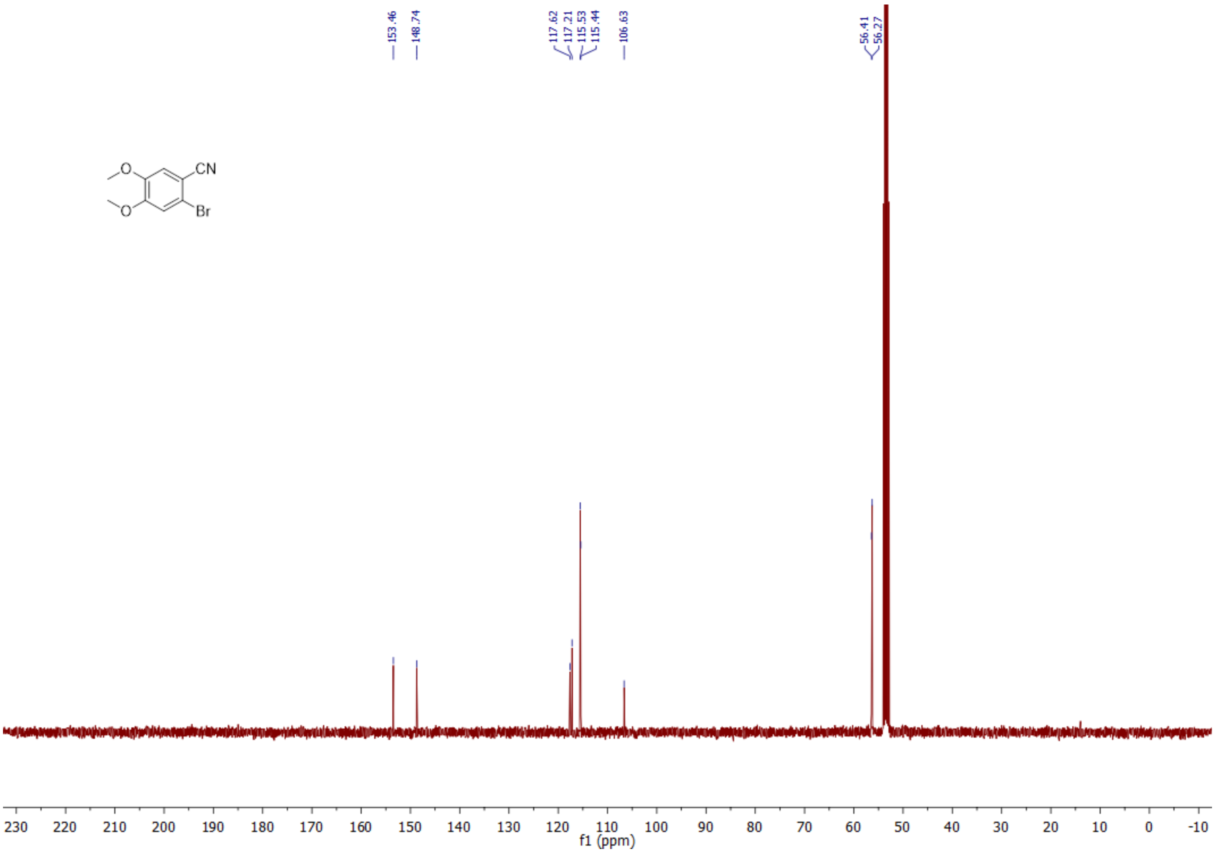

^1H NMR spectrum of compound 6d (400 MHz, methylene chloride-d_2)

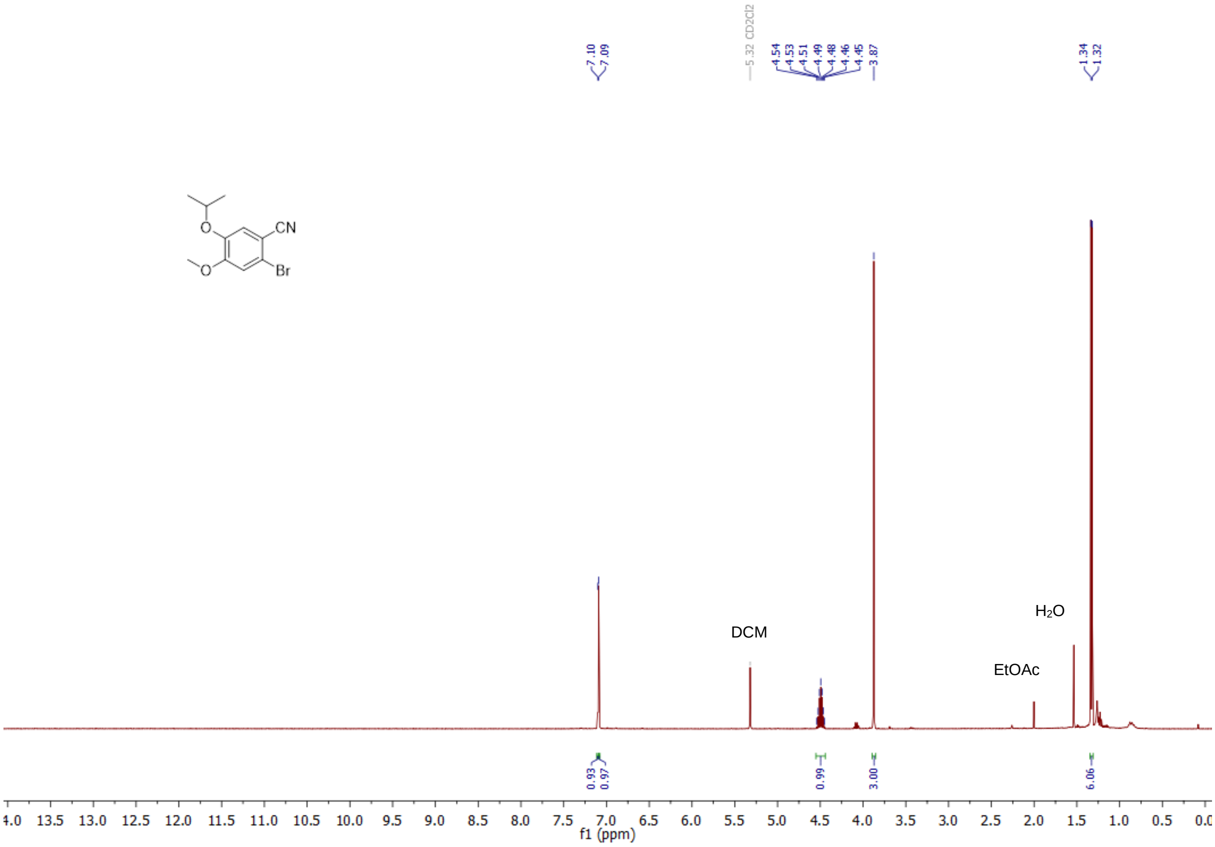

^{13}C NMR spectrum of compound 6d (101 MHz, methylene chloride-d_2)

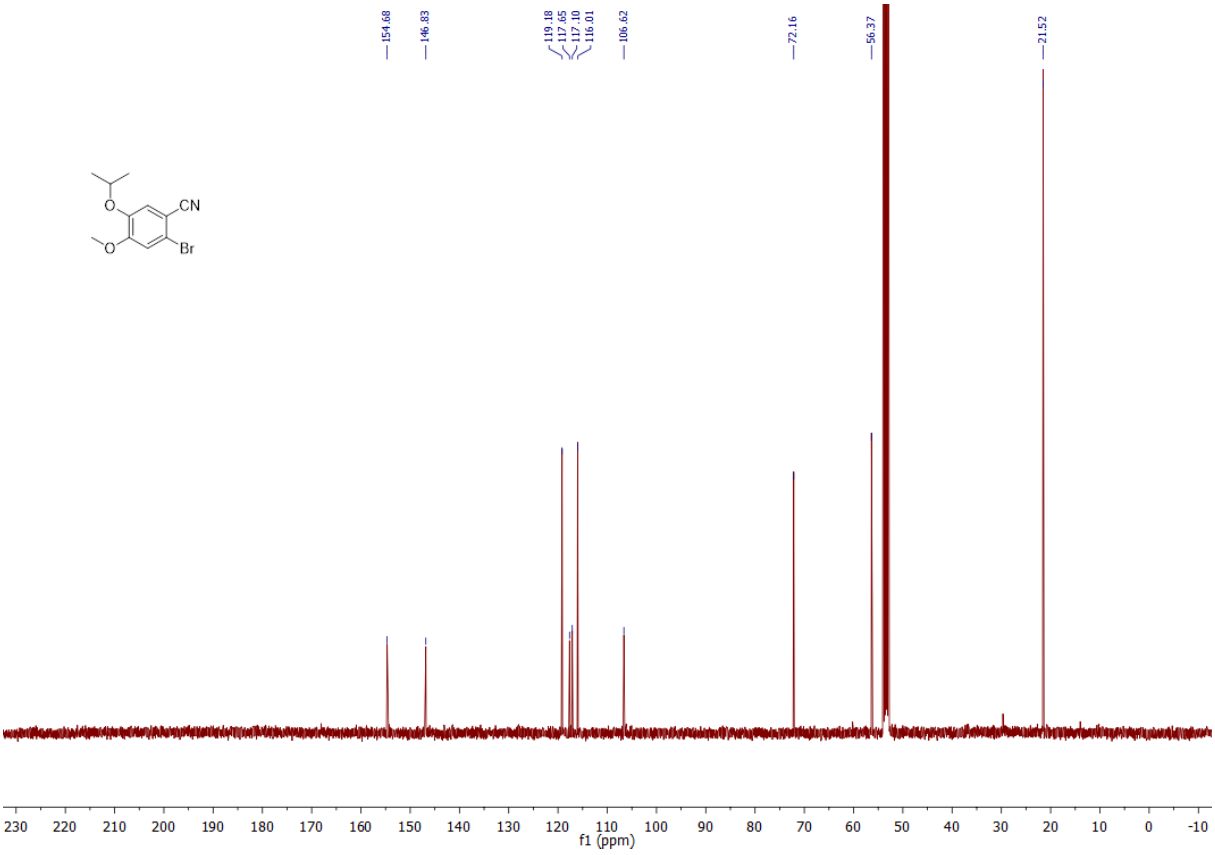

^{1}H NMR spectrum of compound 6e (400 MHz, methylene chloride-d_2)

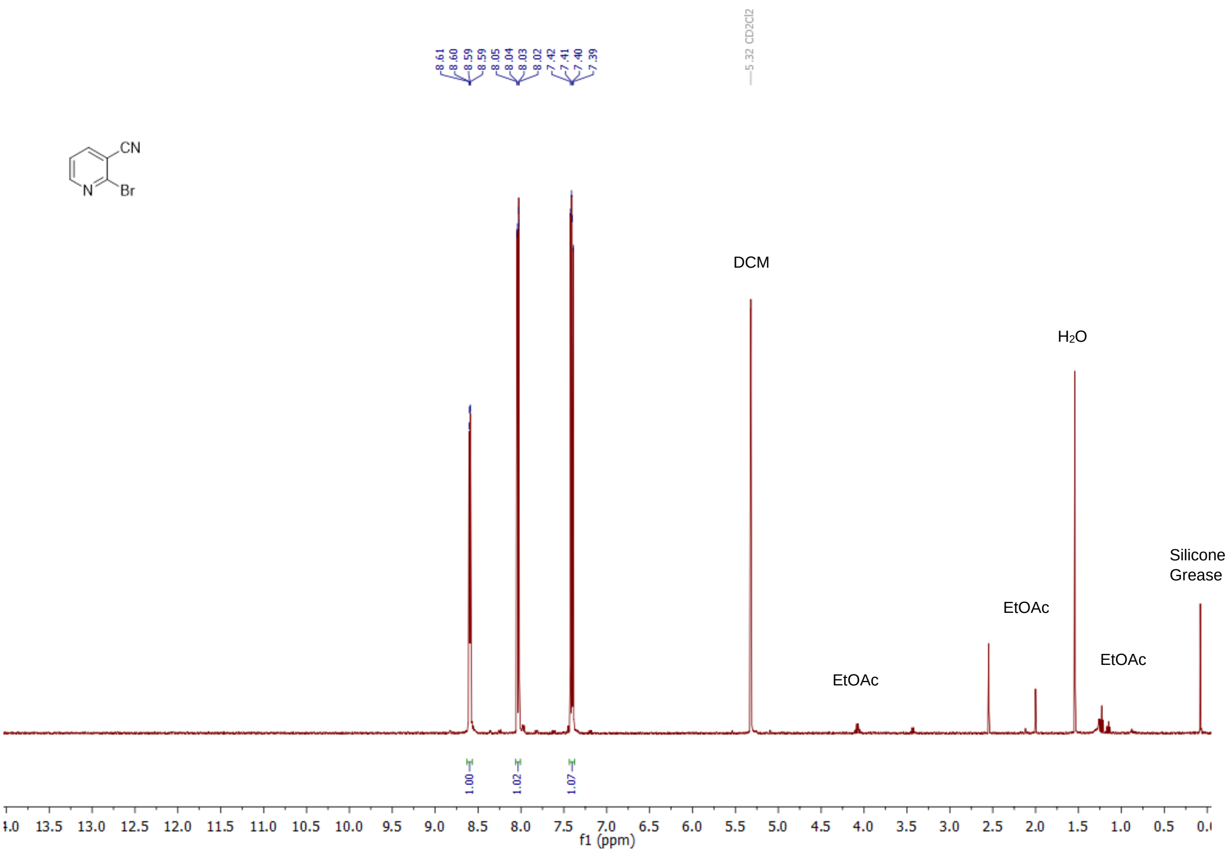

^{13}C NMR spectrum of compound 6e (101 MHz, methylene chloride-d_2)

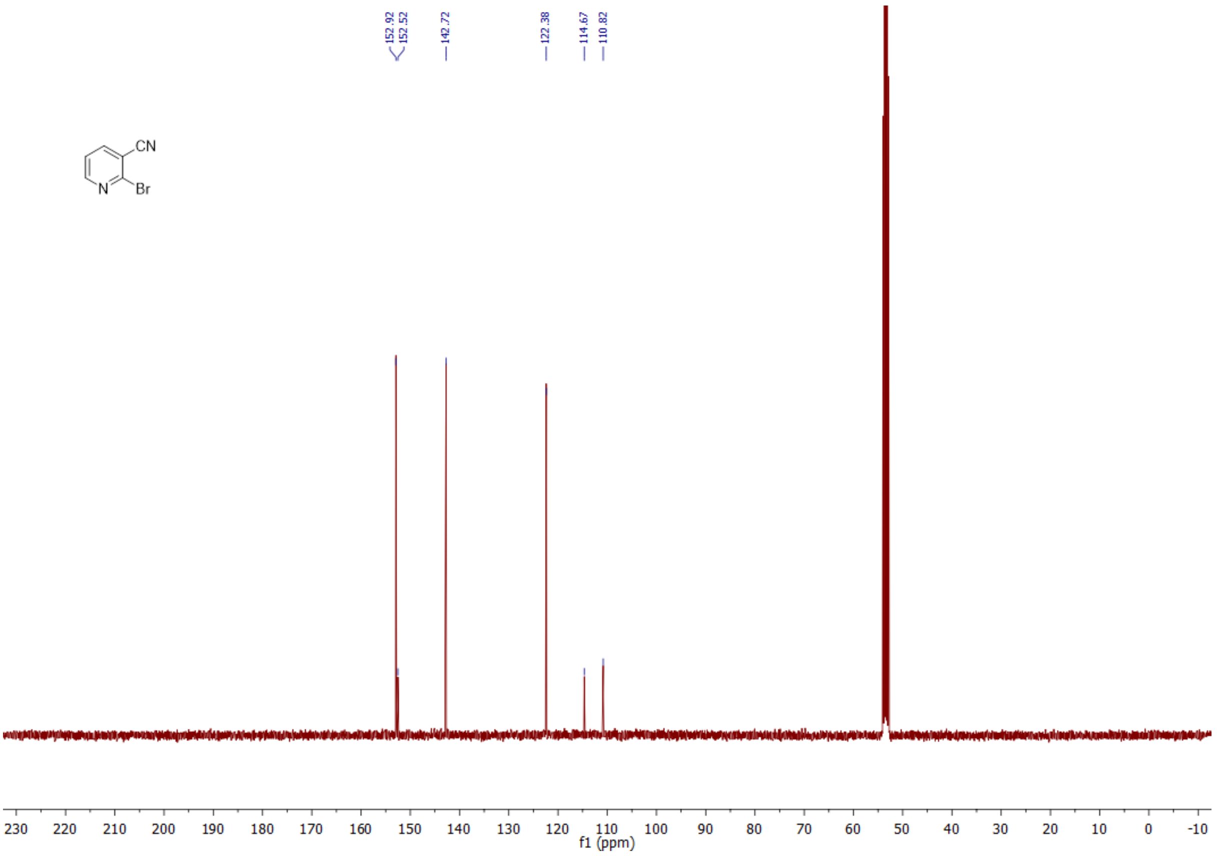

^{1}H NMR spectrum of compound 8a (400 MHz, DMSO-d_6)

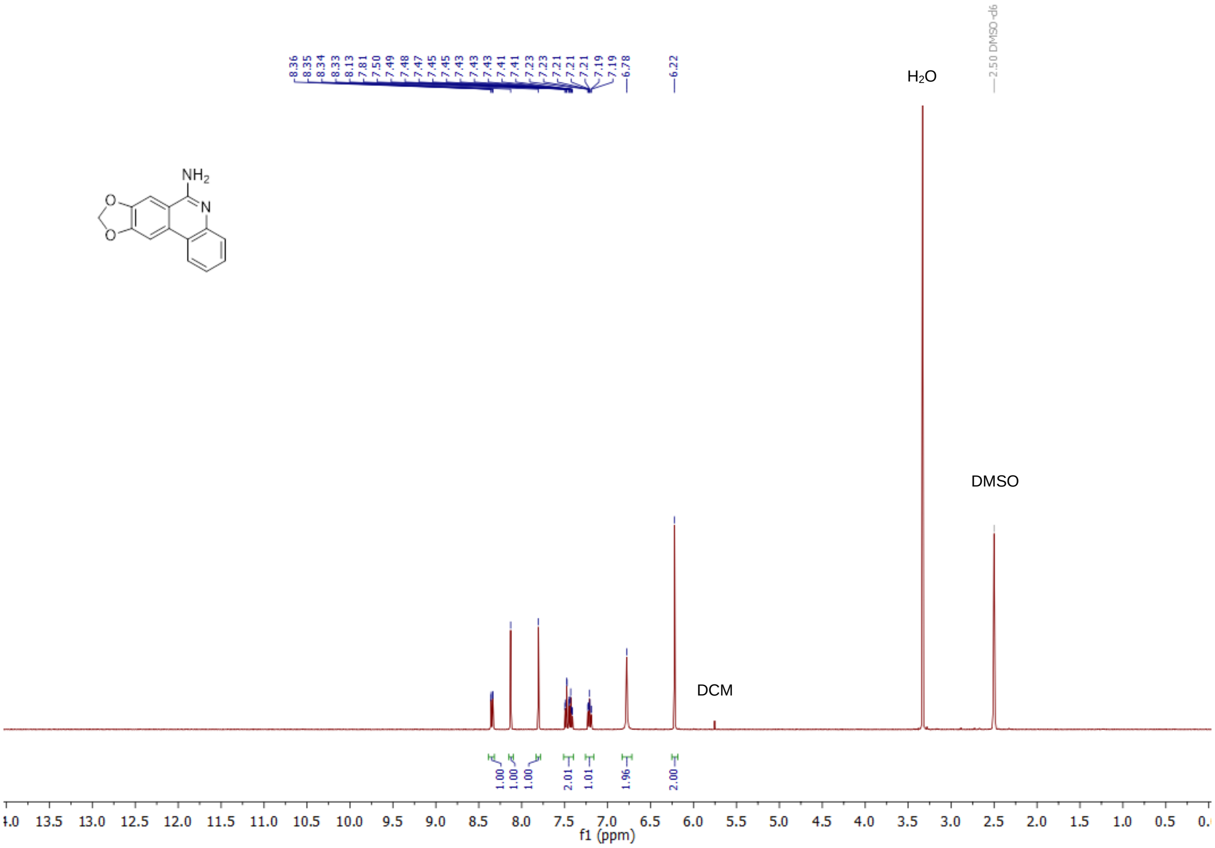

^{13}C NMR spectrum of compound 8a (101 MHz, DMSO-d_6)

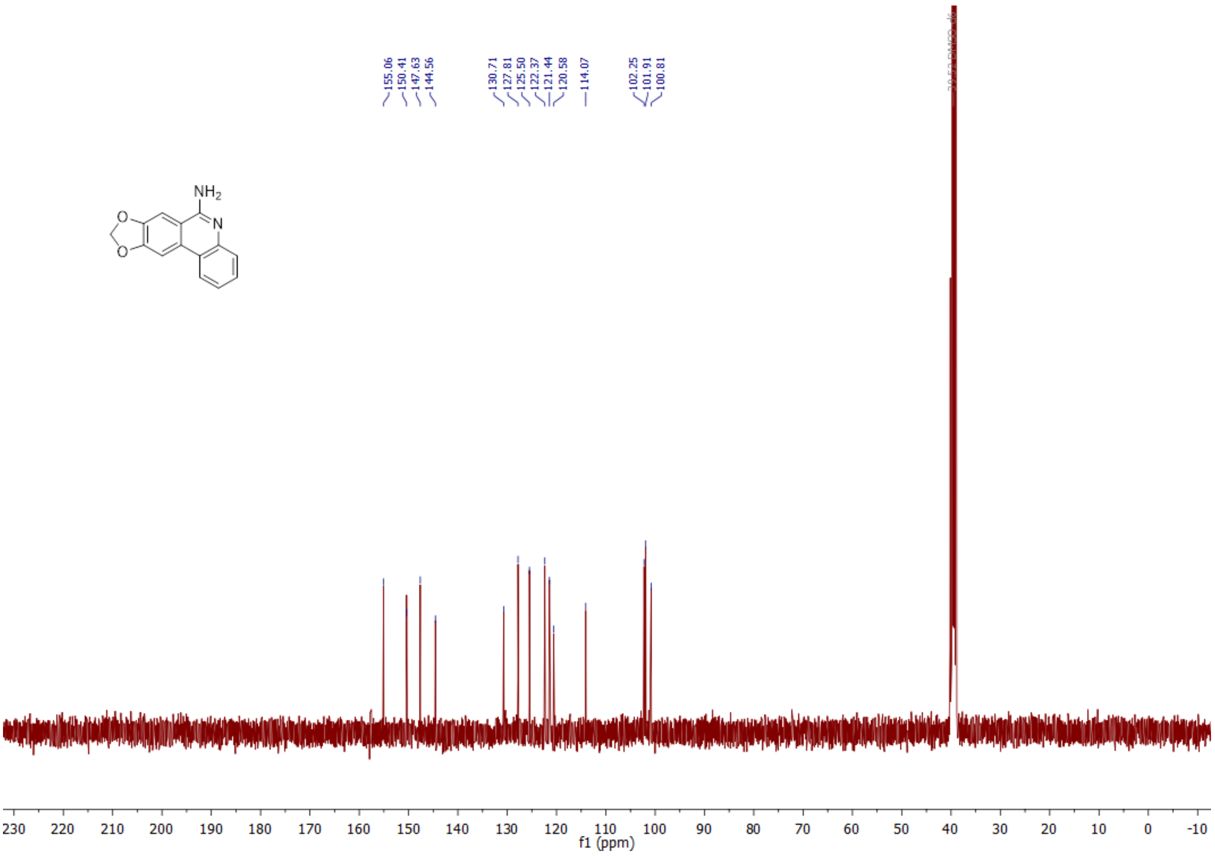

^{1}H NMR spectrum of compound 8b (500 MHz, methylene chloride-d_2)

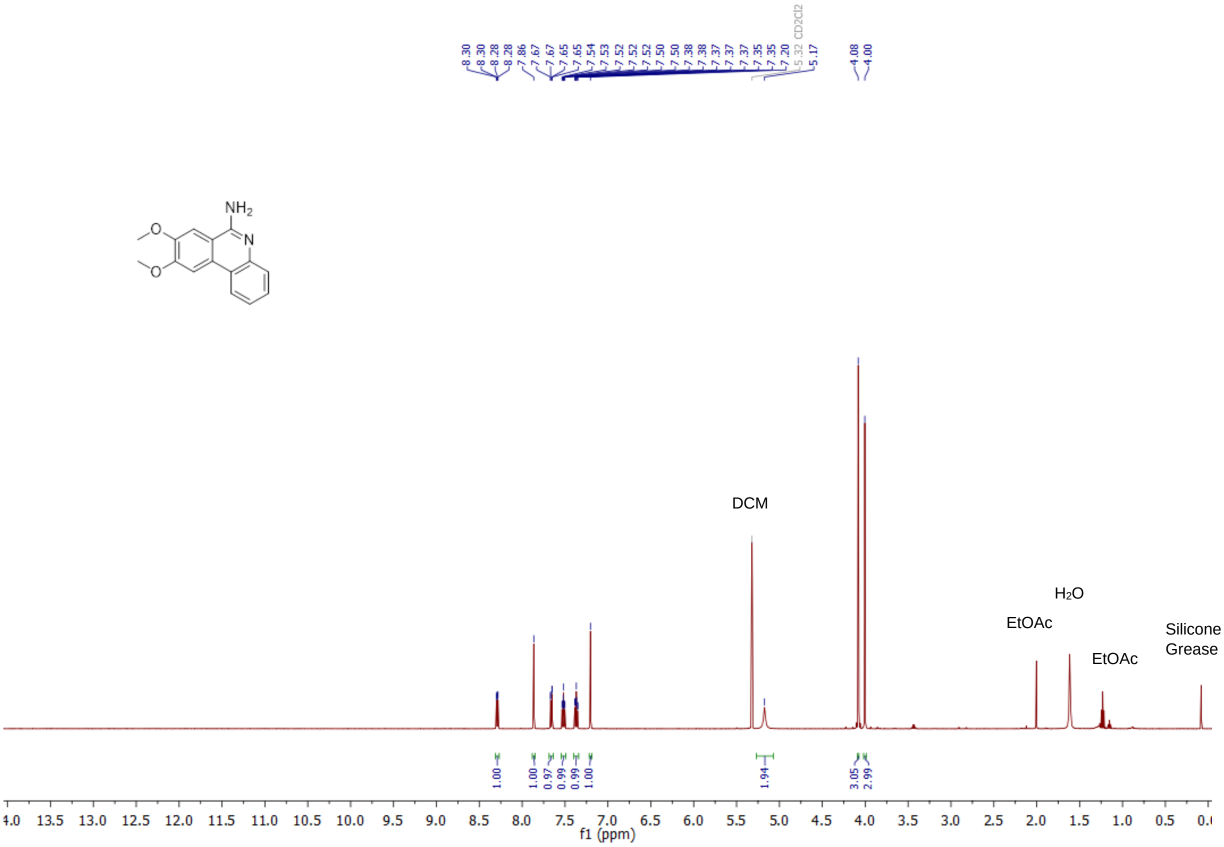

^{13}C NMR spectrum of compound 8b (126 MHz, methylene chloride-d_2)

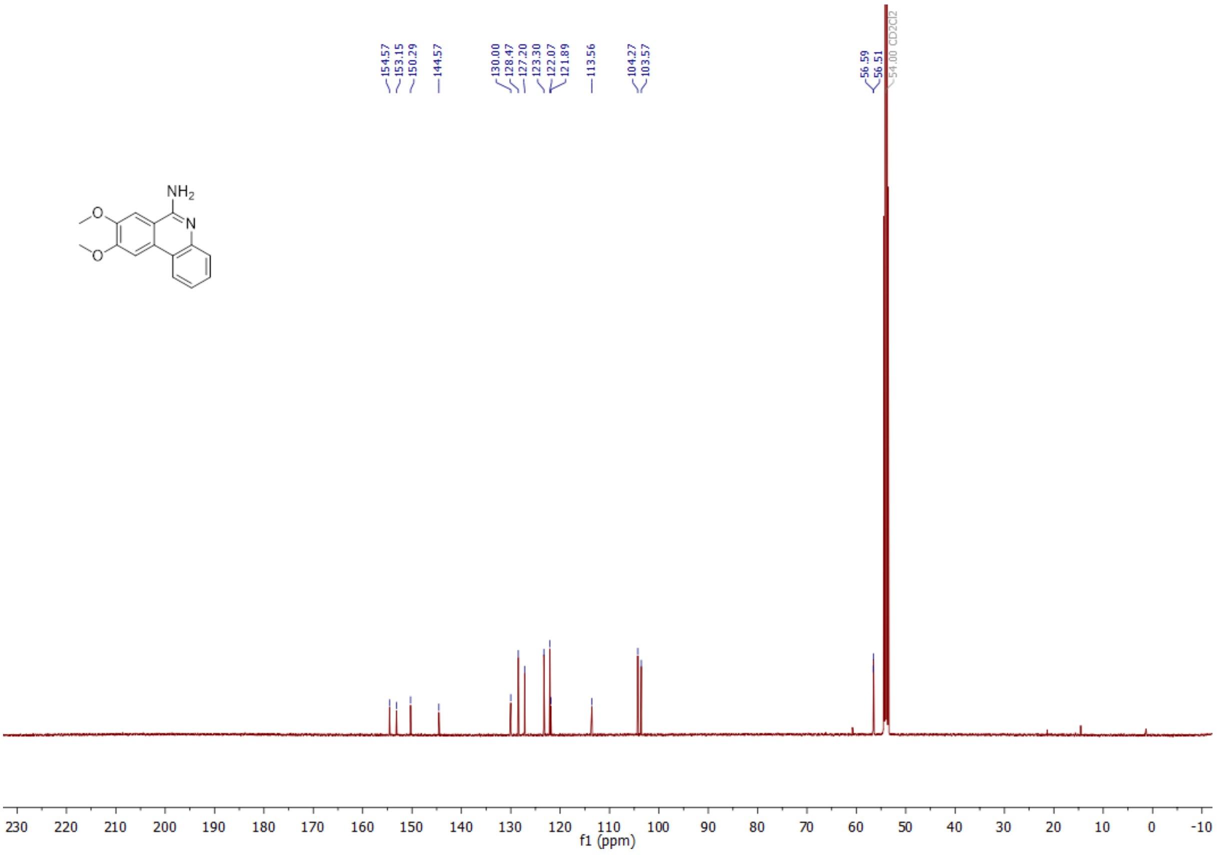

^{1}H NMR spectrum of compound 8c (400 MHz, DMSO-d_6)

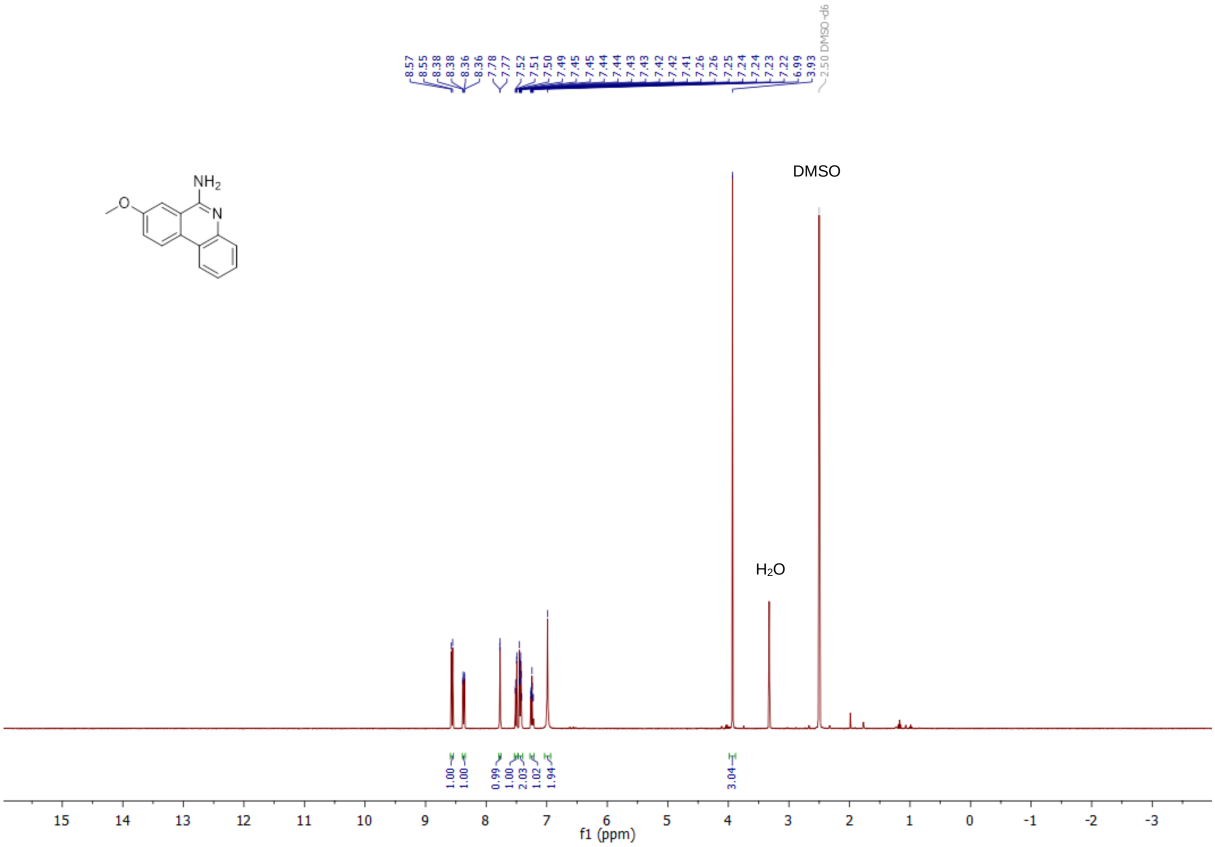

^{13}C NMR spectrum of compound 8c (101 MHz, DMSO-d_6)

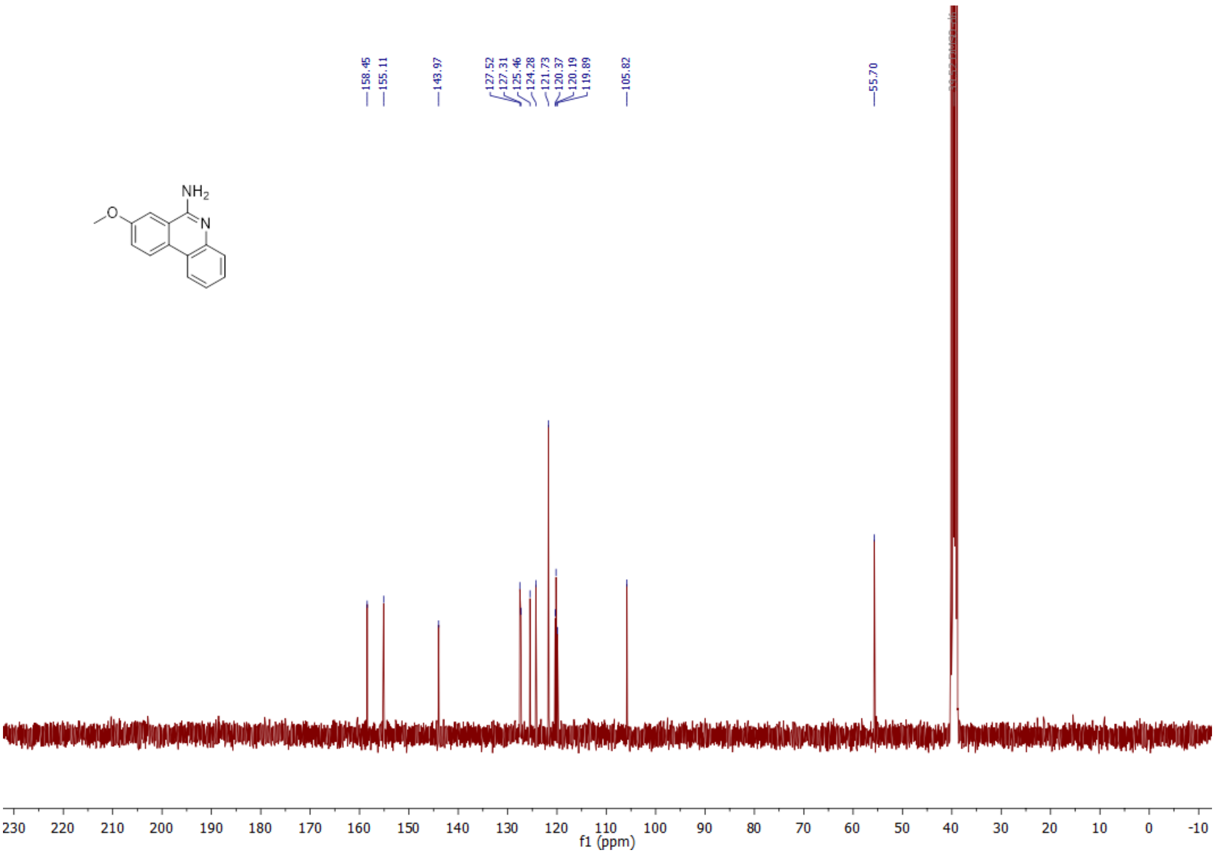

^{1}H NMR spectrum of compound 8d (400 MHz, DMSO-d_6)

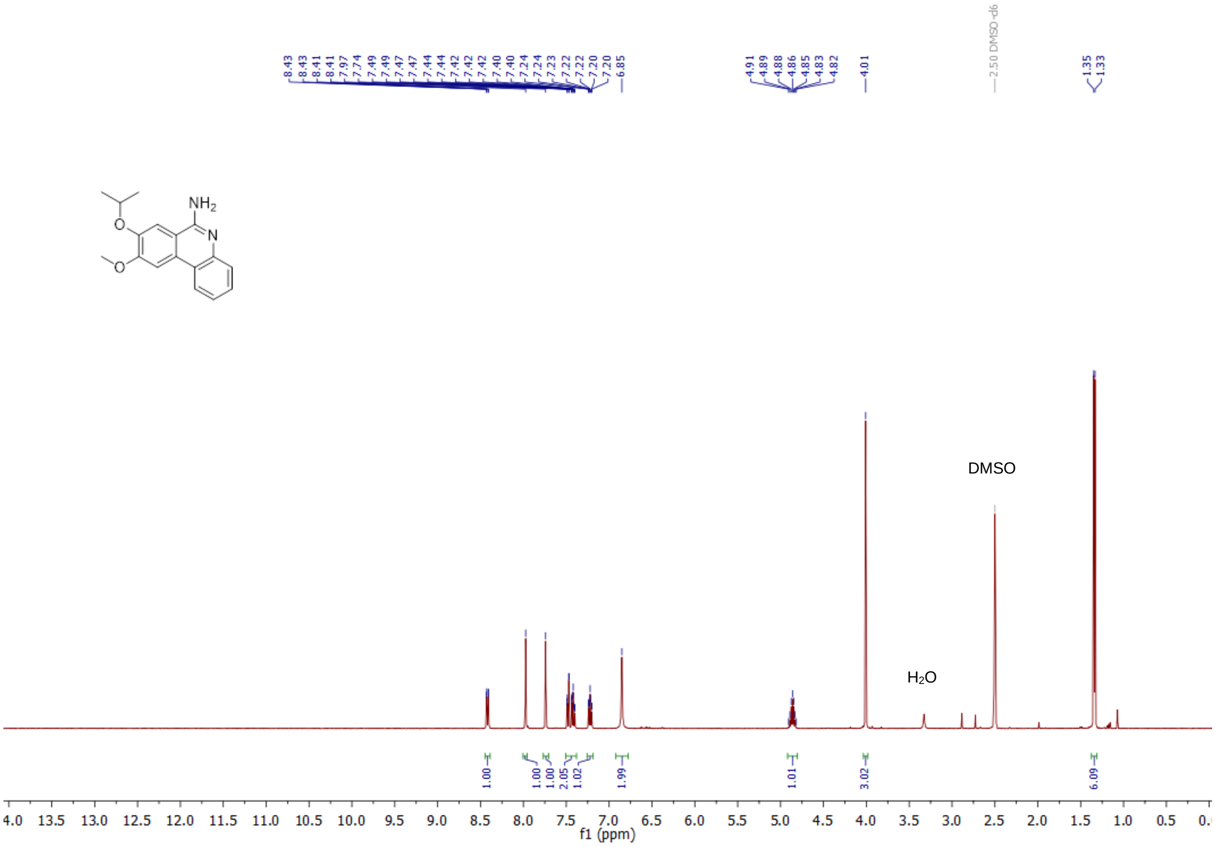

^{13}C NMR spectrum of compound 8d (101 MHz, DMSO-d_6)

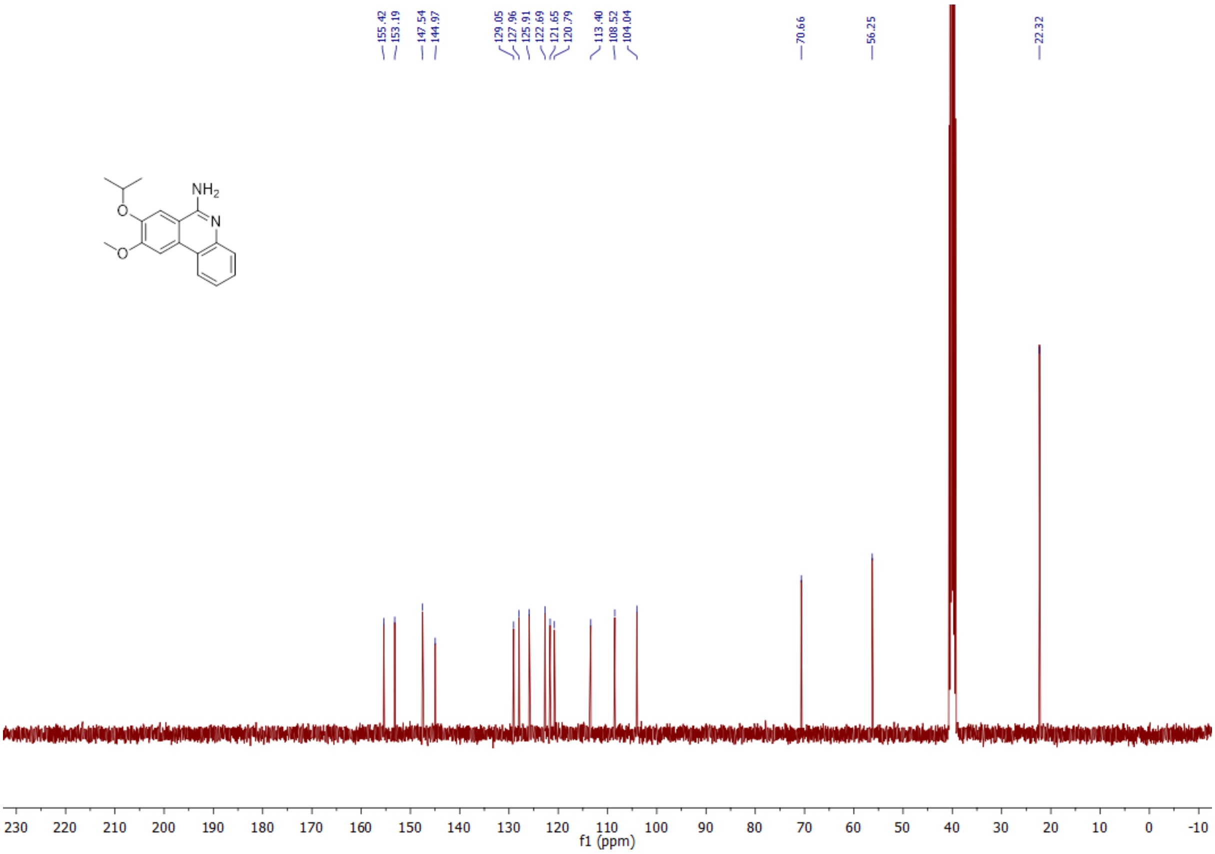

^{1}H NMR spectrum of compound 8e (400 MHz, DMSO-d_6)

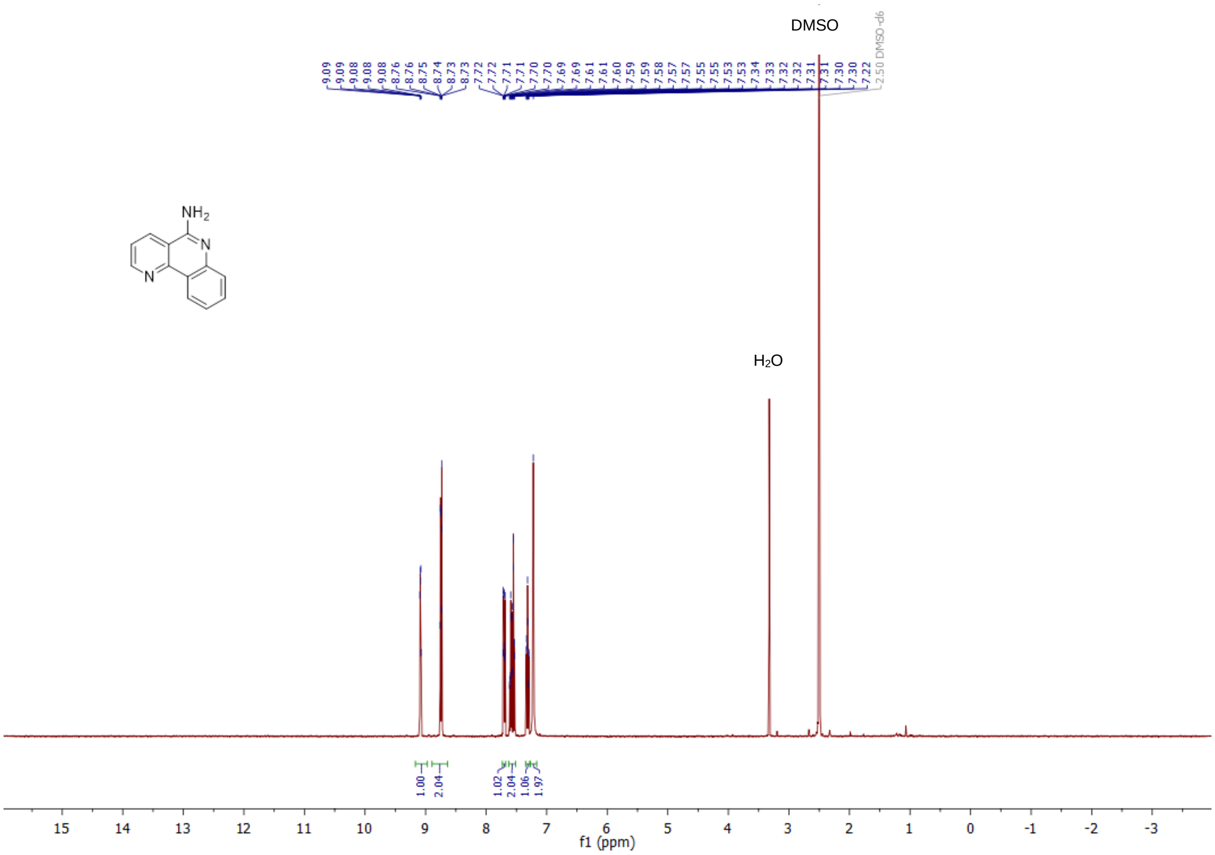

^{13}C NMR spectrum of compound 8e (101 MHz, DMSO-d_6)

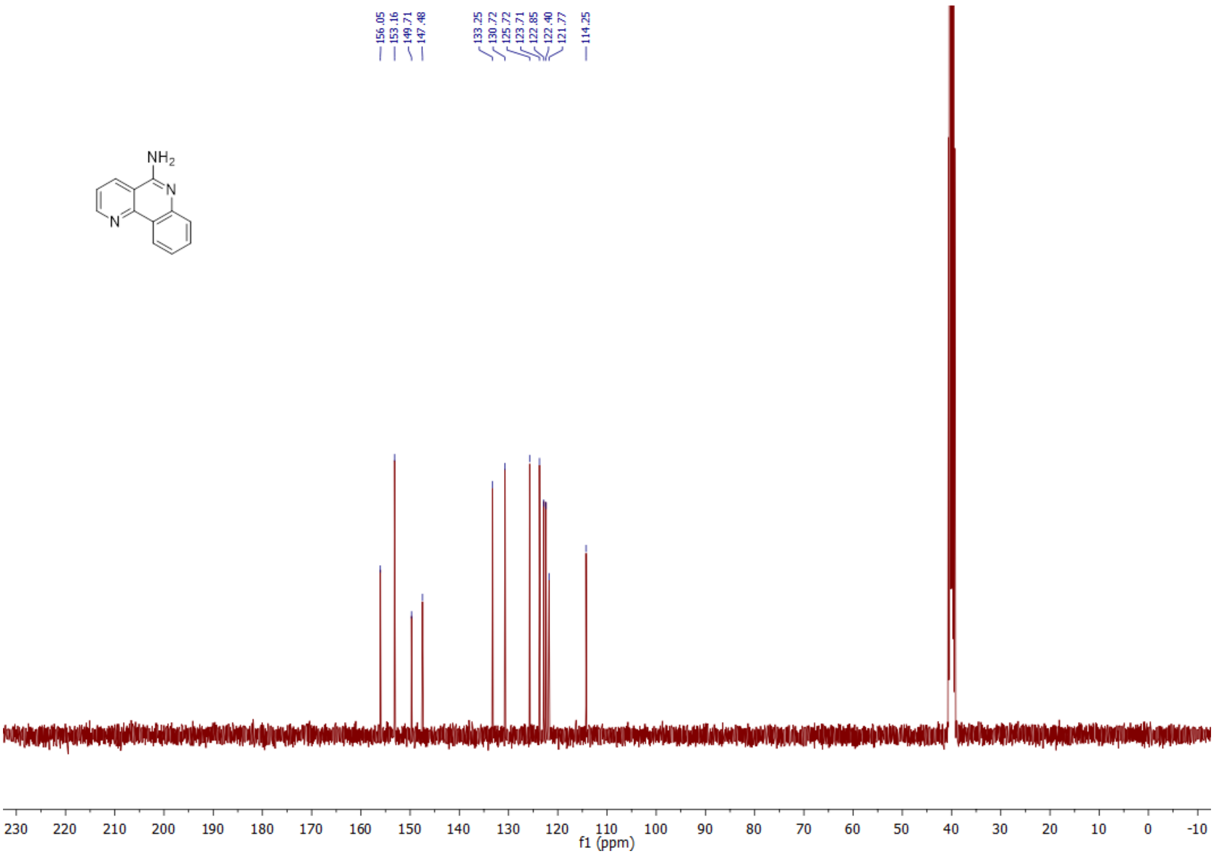

^{1}H NMR spectrum of compound 3 (400 MHz, $CDCl_3$)

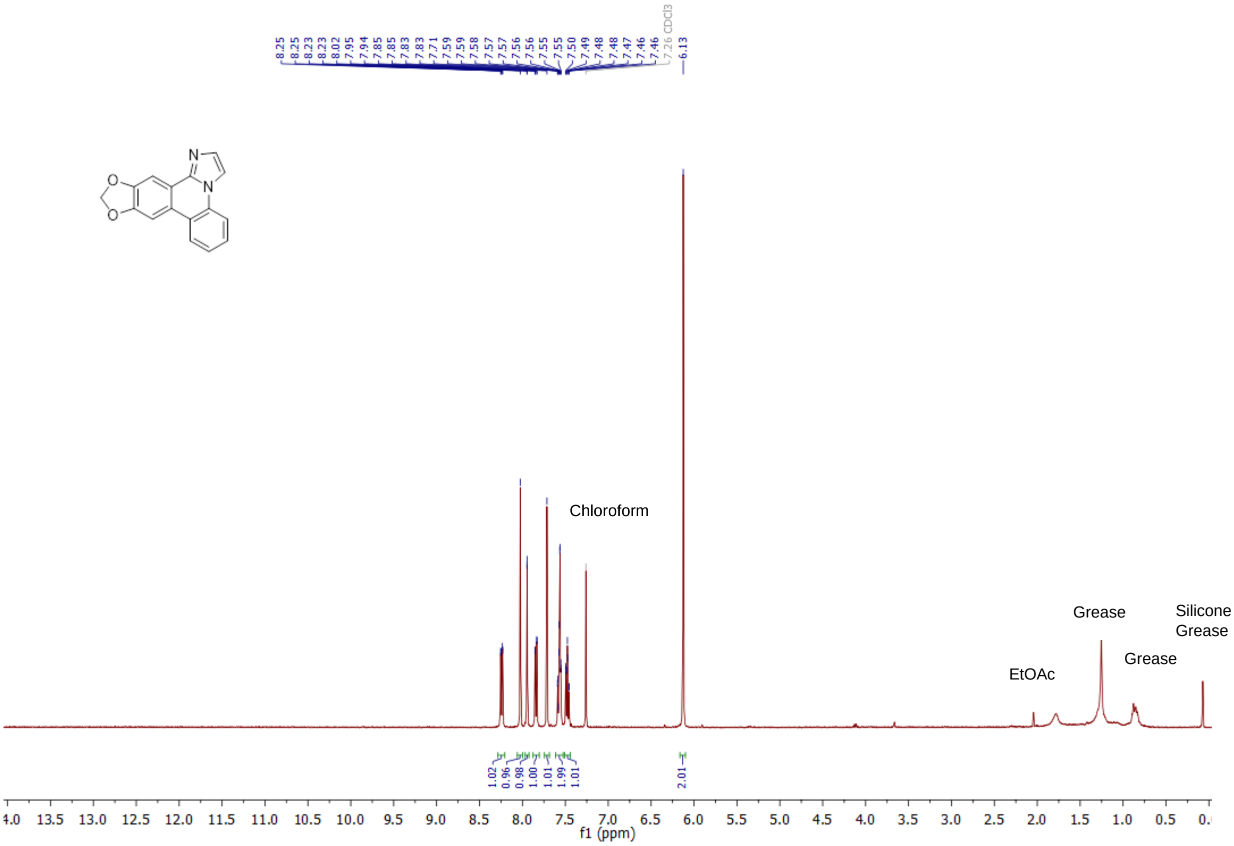

^{13}C NMR spectrum of compound 3 (101 MHz, $CDCl_3$)

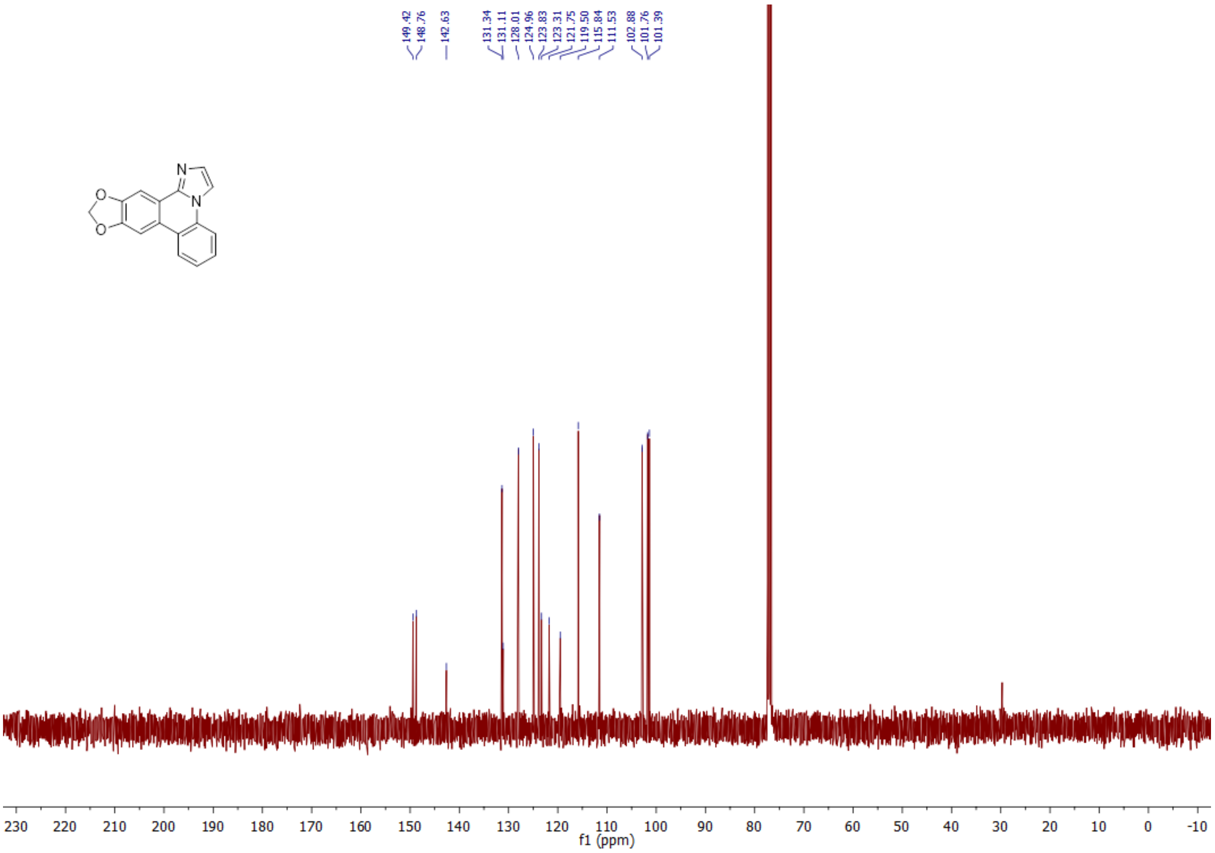

^{1}H NMR spectrum of compound 3 (400 MHz, MeOD)

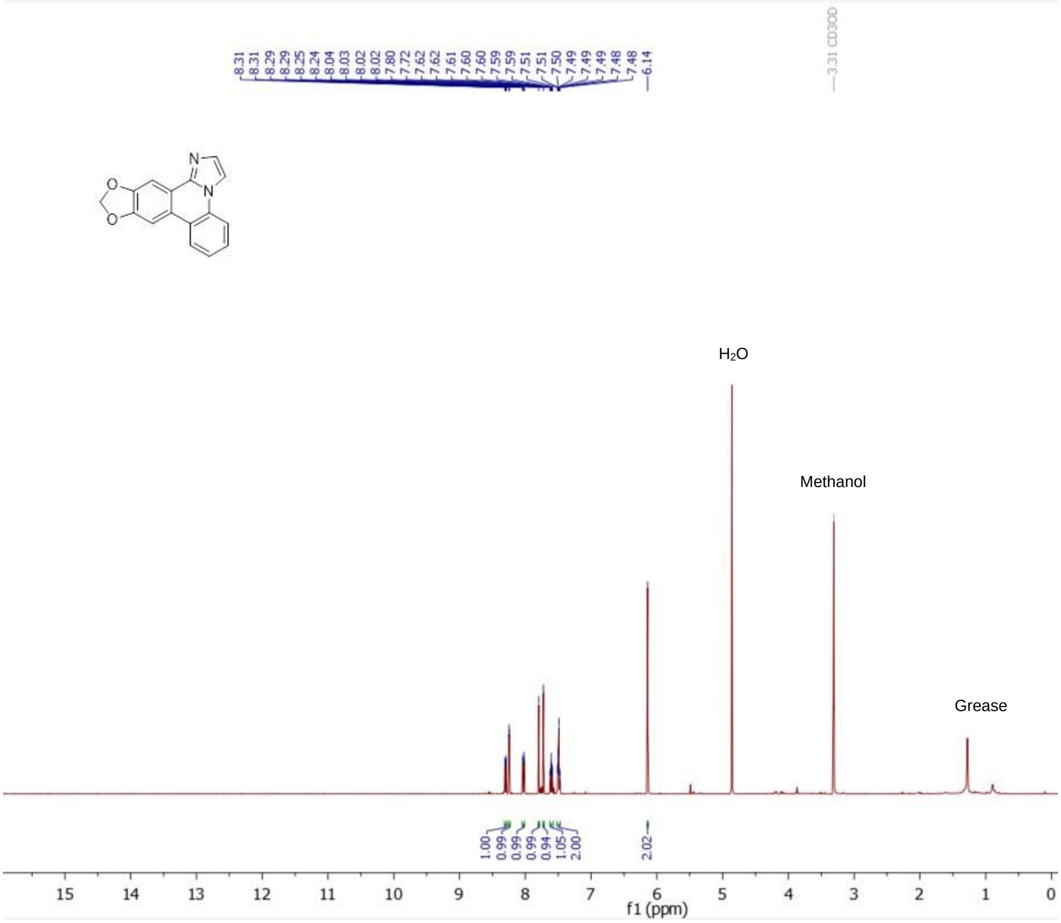

^{13}C NMR spectrum of compound 3 (101 MHz, MeOD)

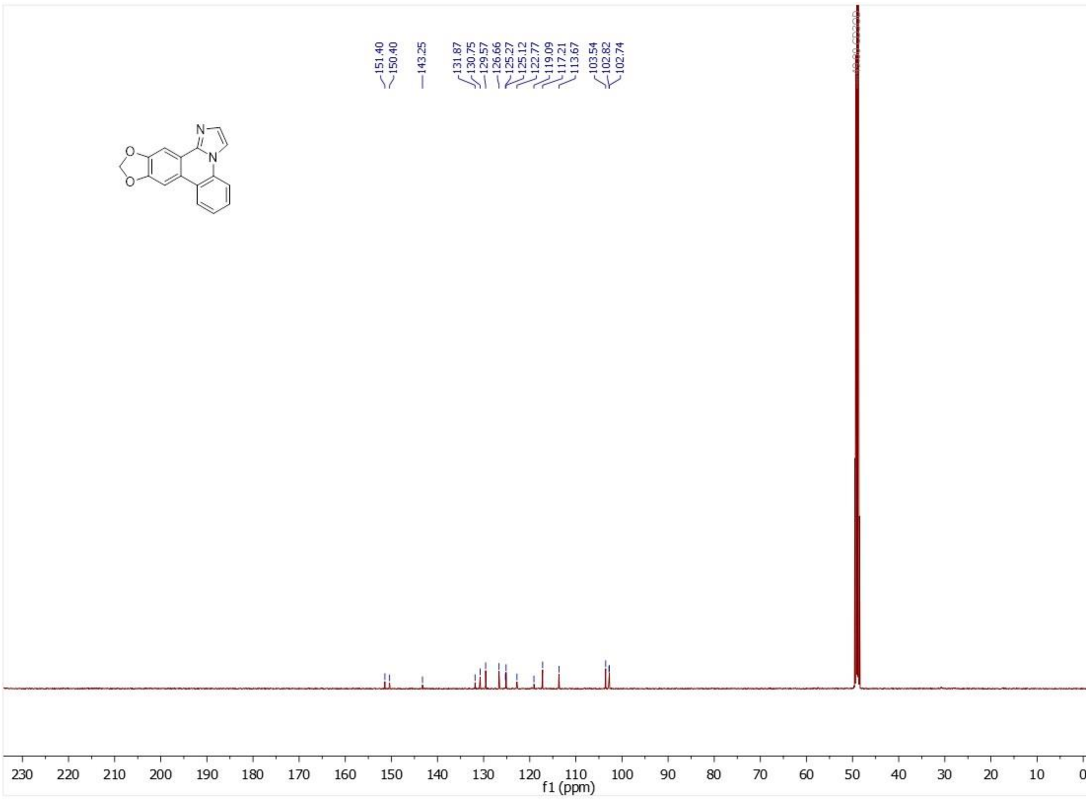

^{1}H NMR spectrum of compound 9 (400 MHz, methylene chloride-d_2)

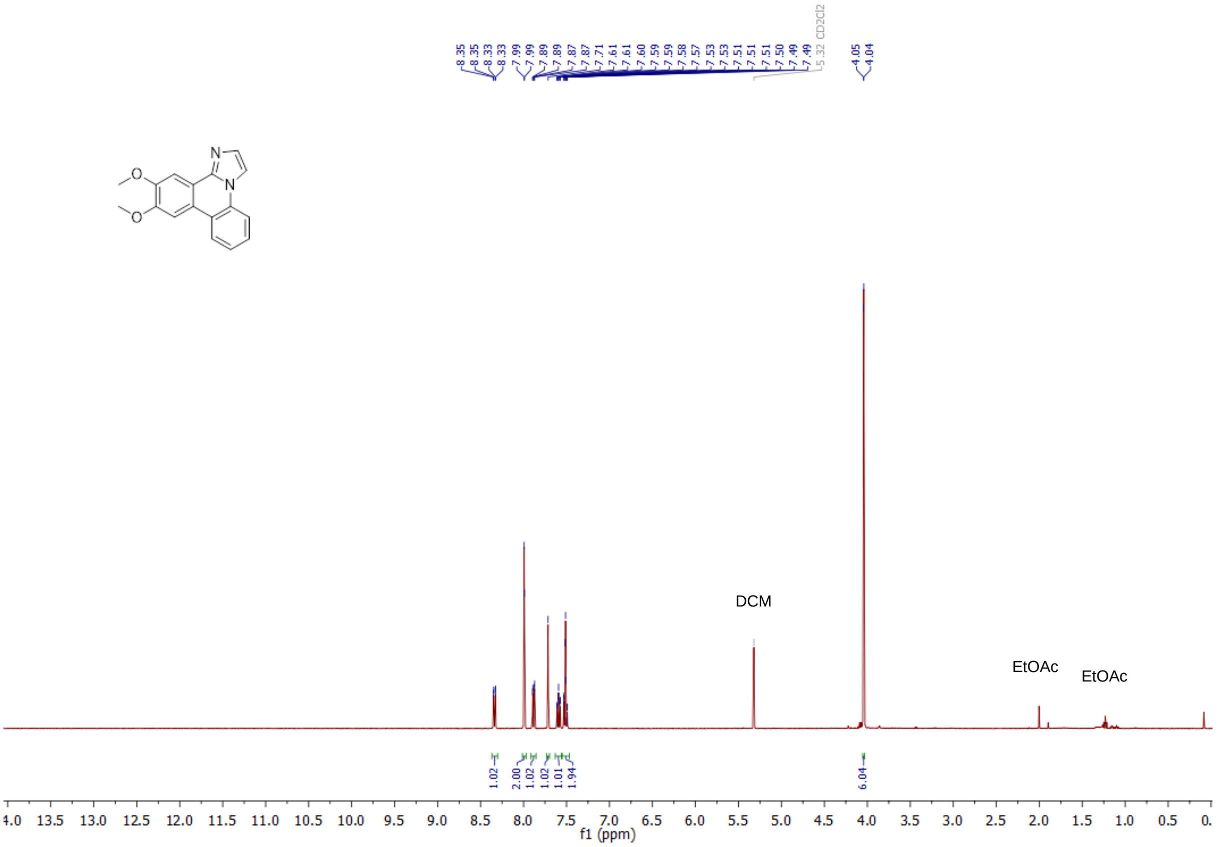

^{13}C NMR spectrum of compound 9 (101 MHz, methylene chloride-d_2)

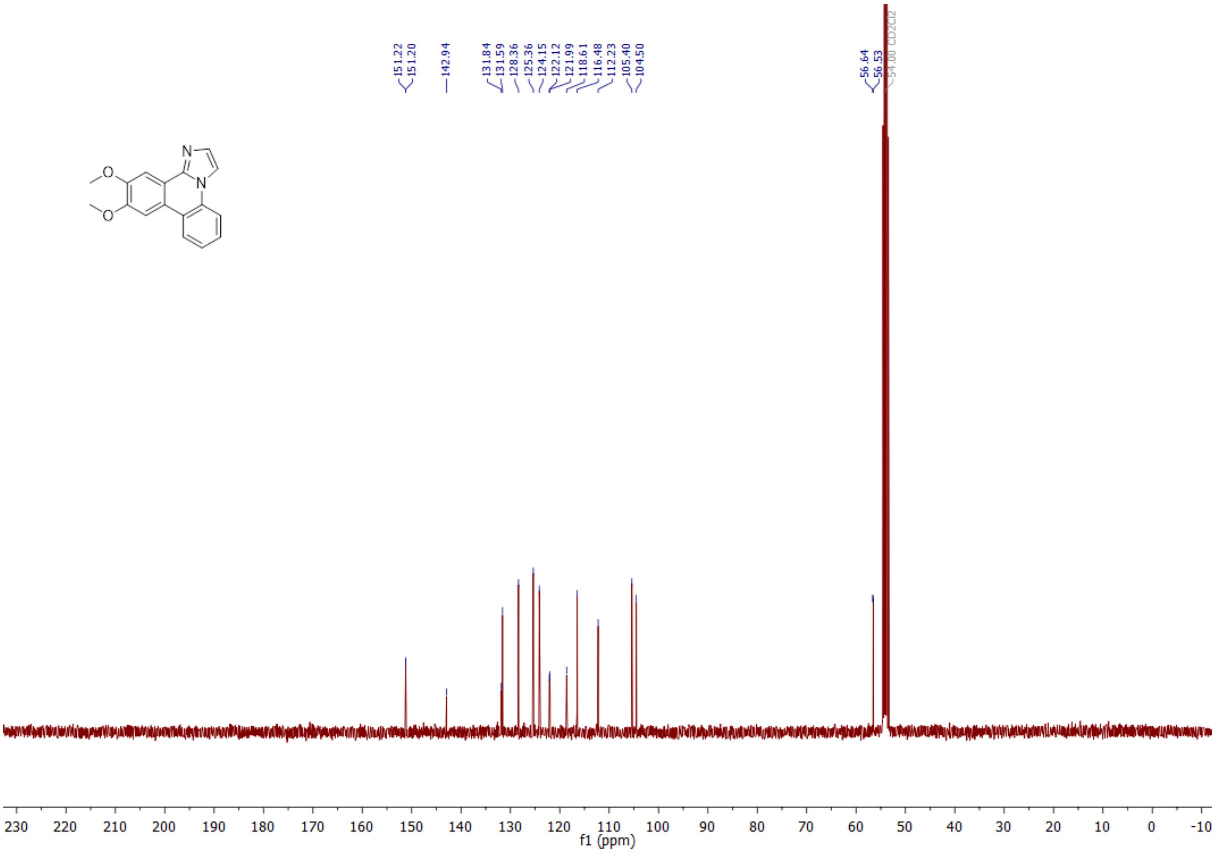

^{1}H NMR spectrum of compound 10 (400 MHz, methylene chloride-d_2)

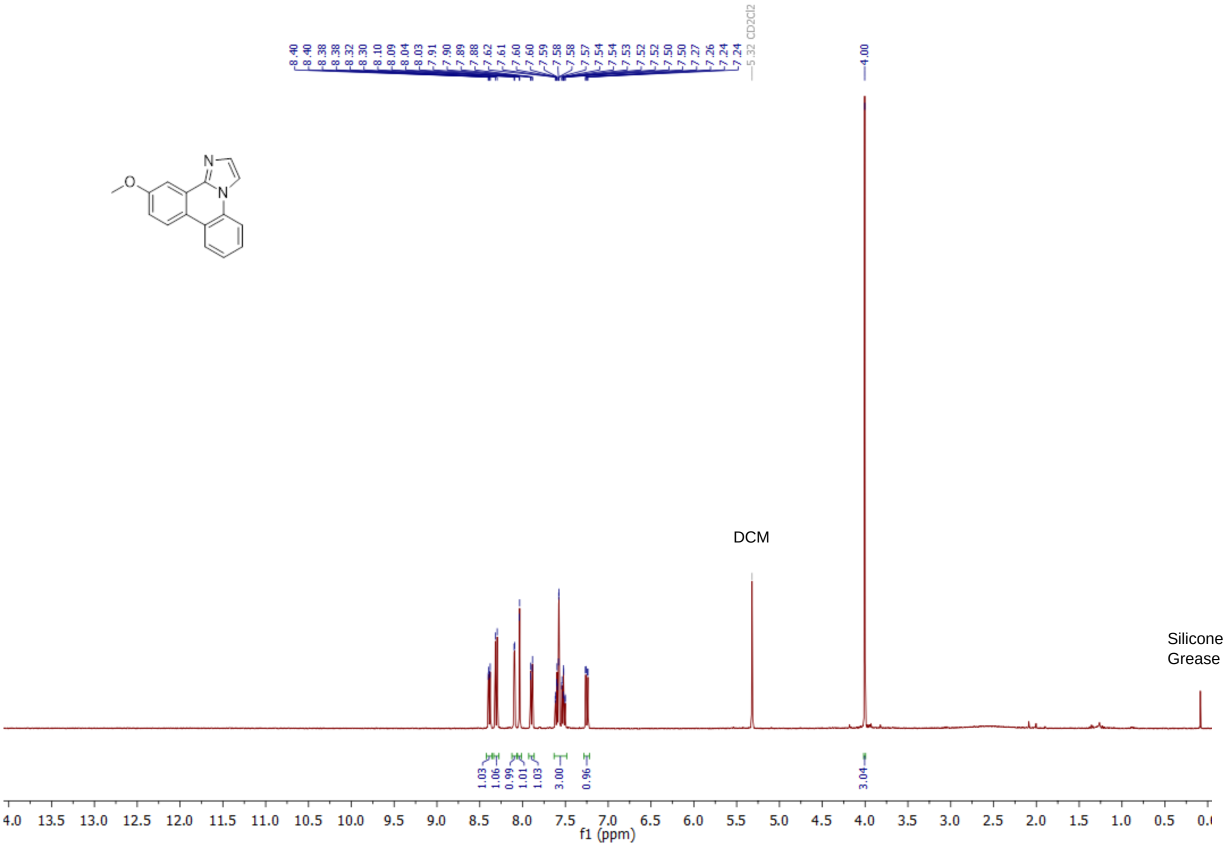

^{13}C NMR spectrum of compound 10 (101 MHz, methylene chloride-d_2)

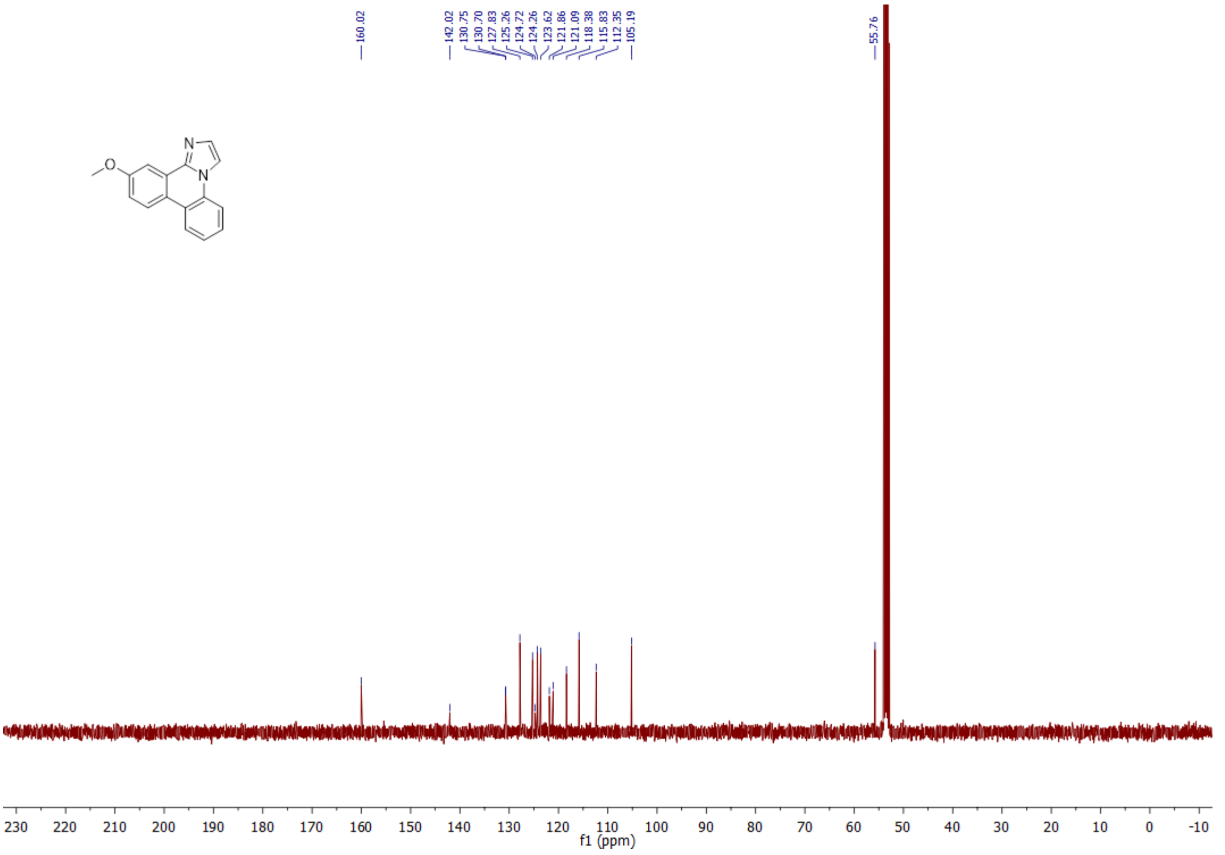

^{1}H NMR spectrum of compound 11 (400 MHz, methylene chloride-d_2)

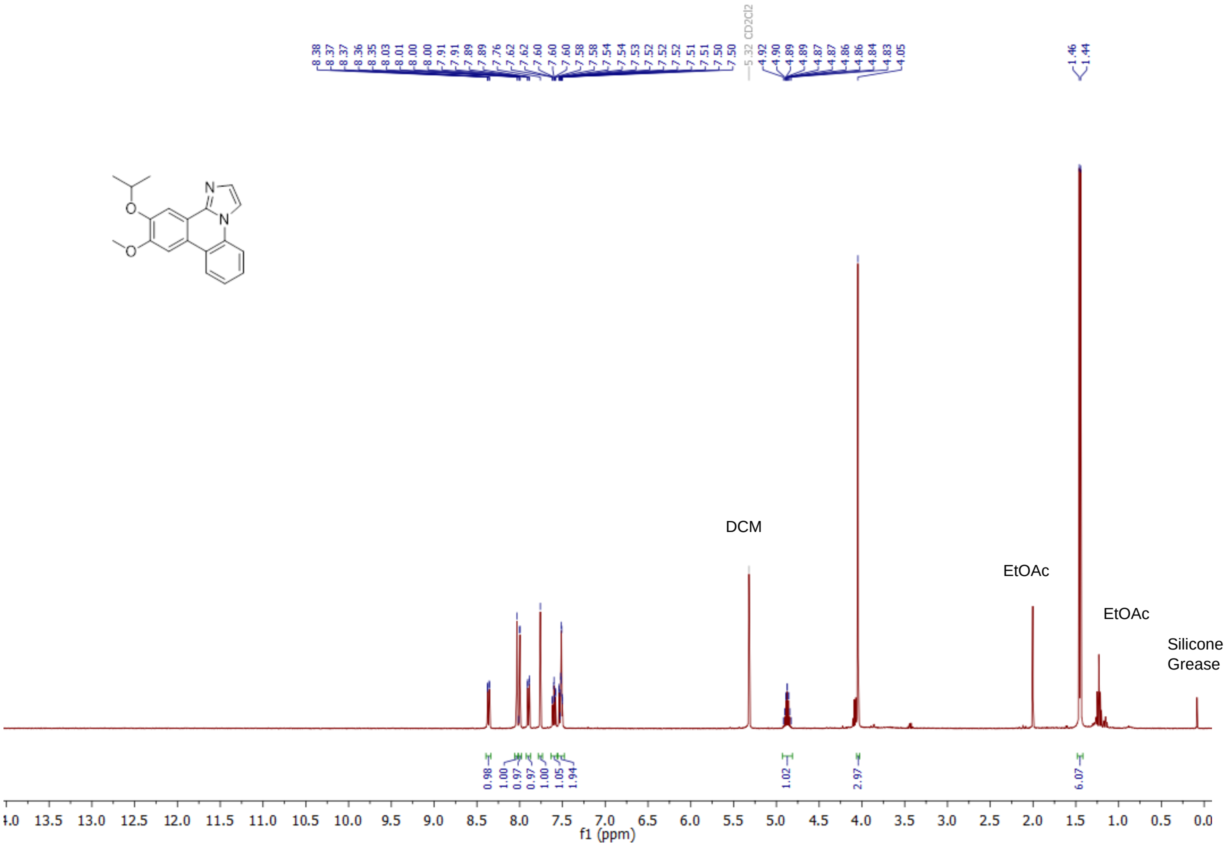

^{13}C NMR spectrum of compound 11 (101 MHz, methylene chloride-d_2)

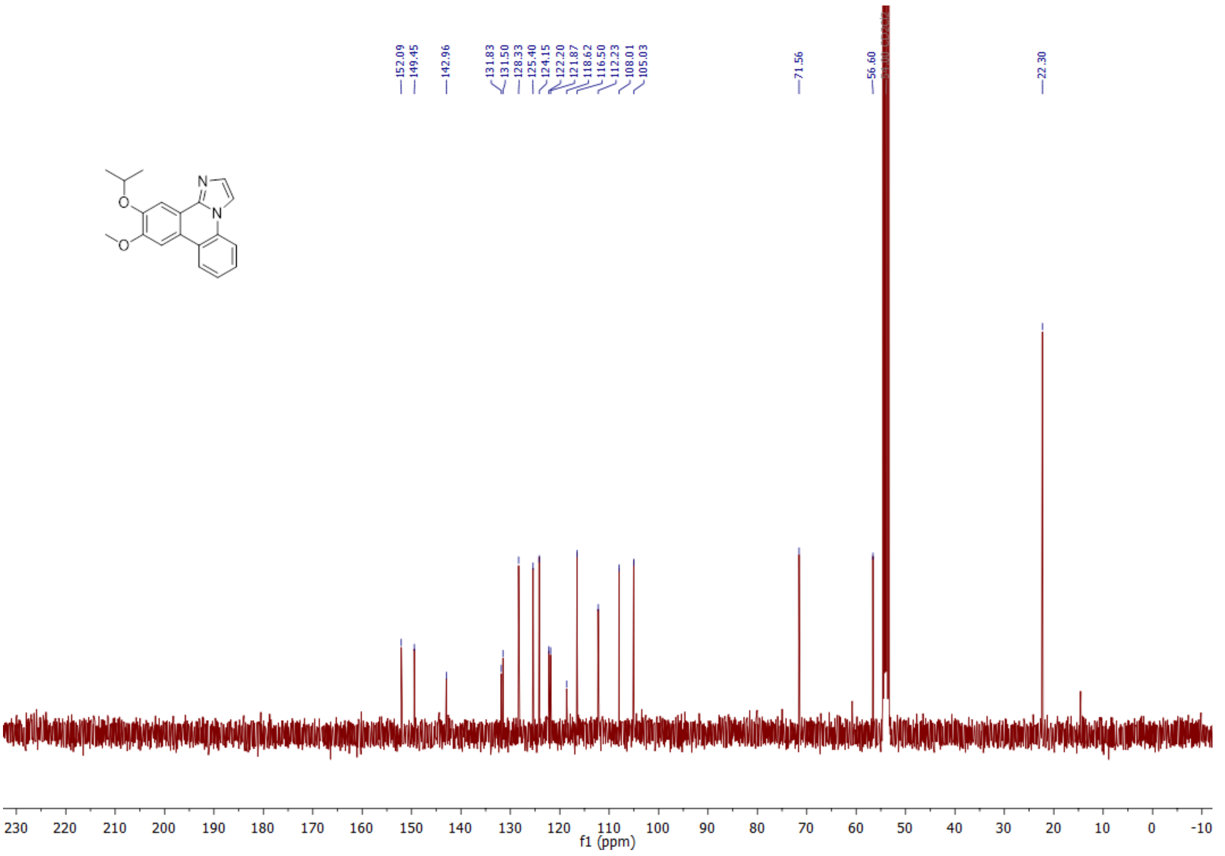

^{1}H NMR spectrum of compound 12 (400 MHz, methylene chloride-d_2)

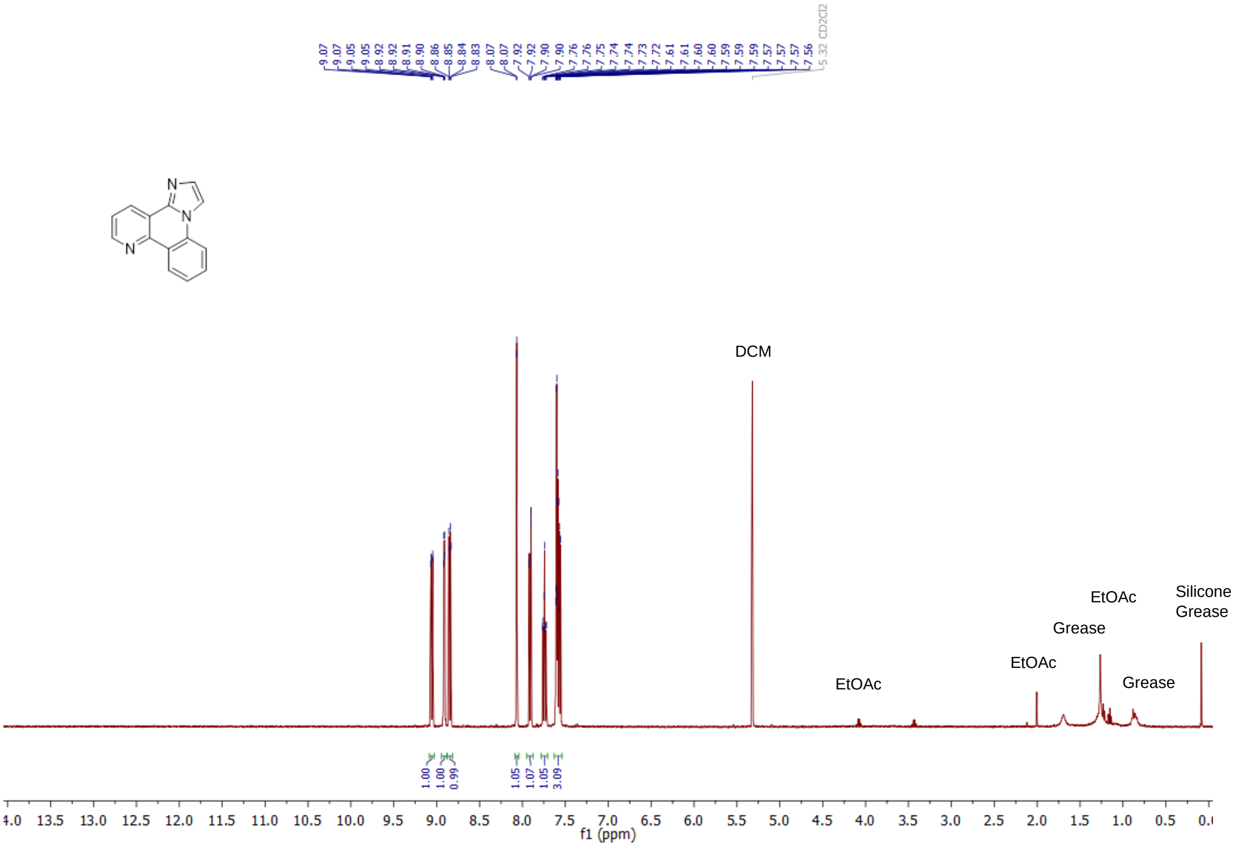

^{13}C NMR spectrum of compound 12 (101 MHz, methylene chloride-d_2)

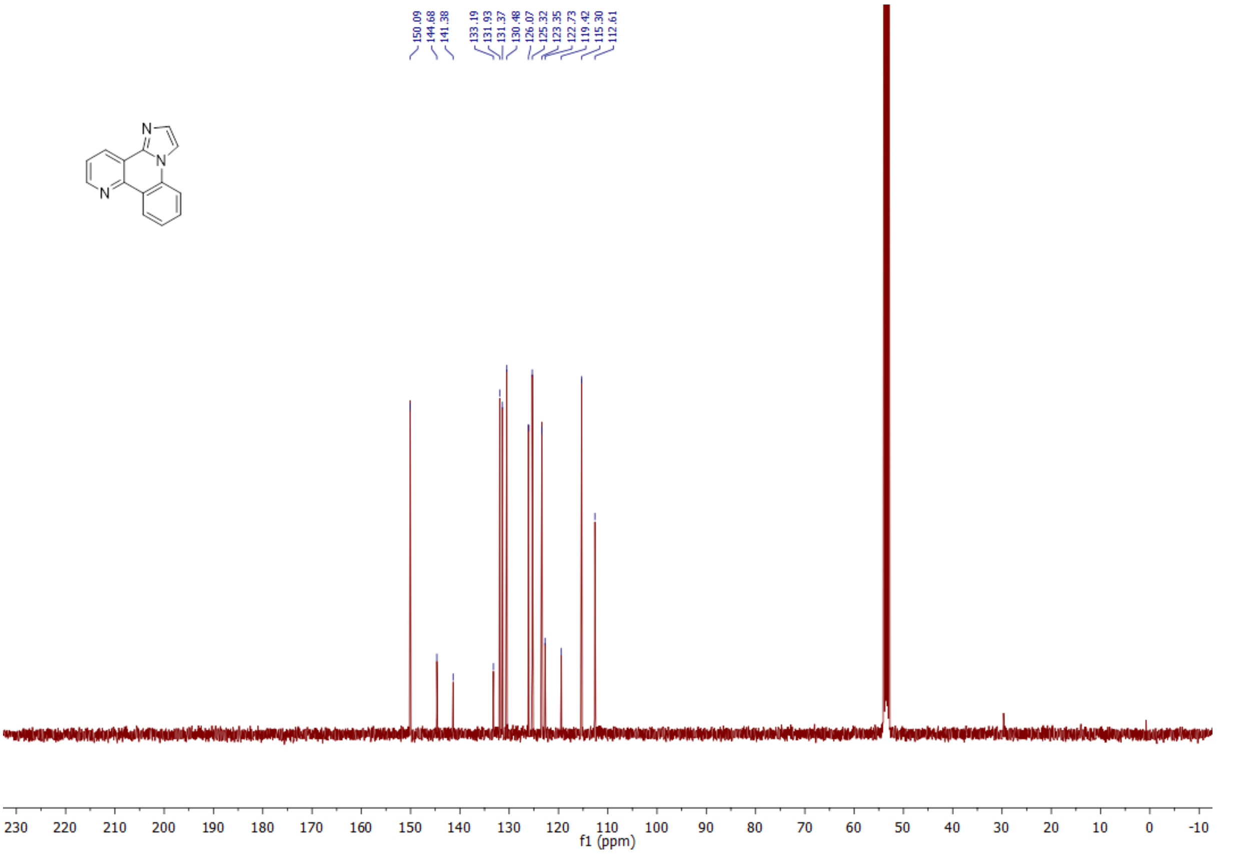

^{1}H NMR spectrum of compound 13 (400 MHz, methylene chloride-d_2)

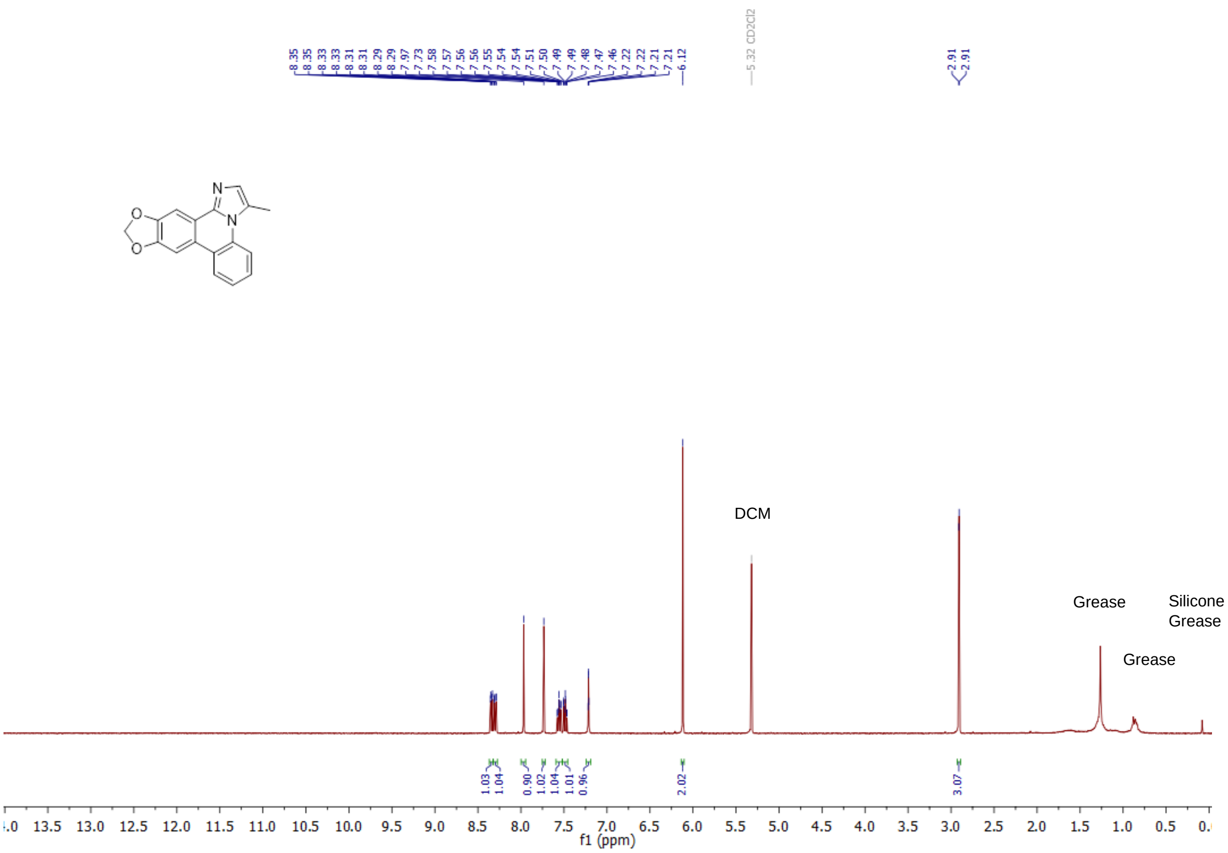

^{13}C NMR spectrum of compound 13 (101 MHz, methylene chloride-d_2)

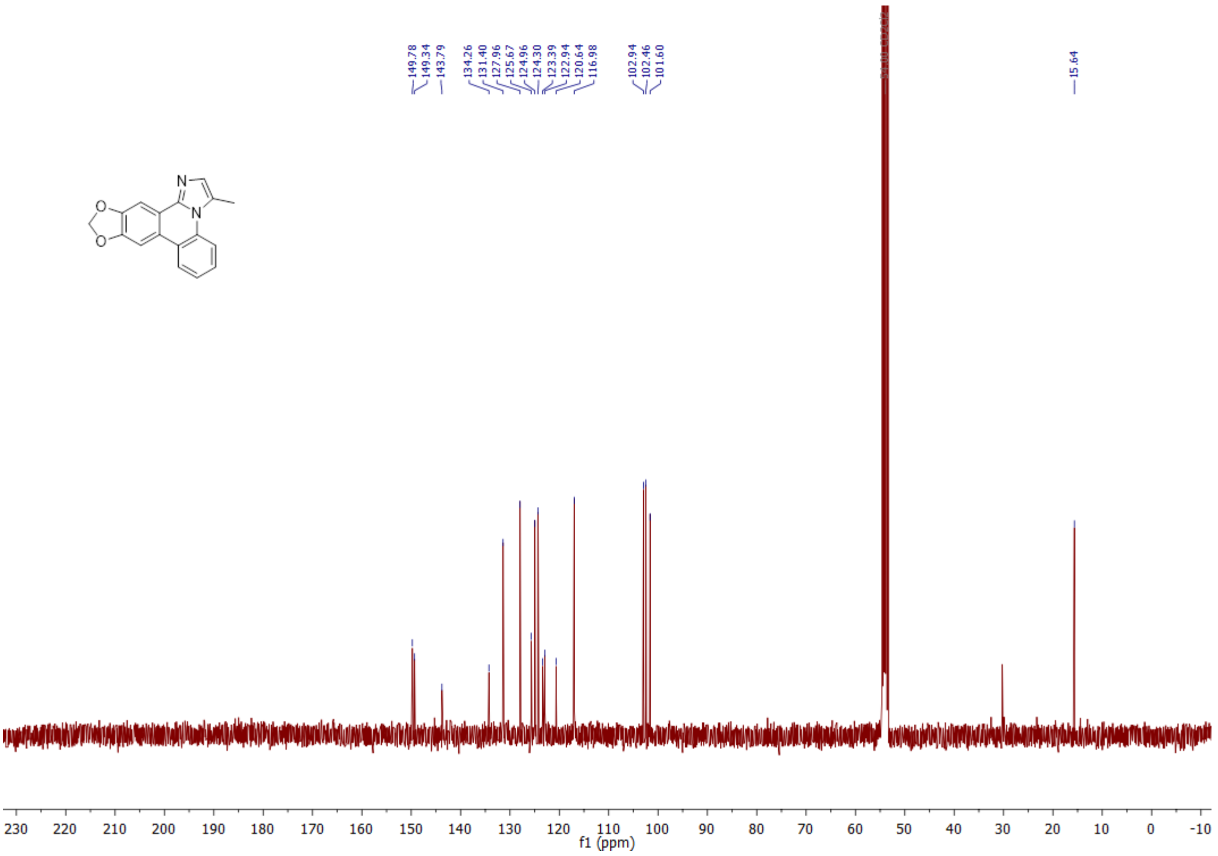

^{1}H NMR spectrum of compound 14 (400 MHz, methylene chloride-d_2)

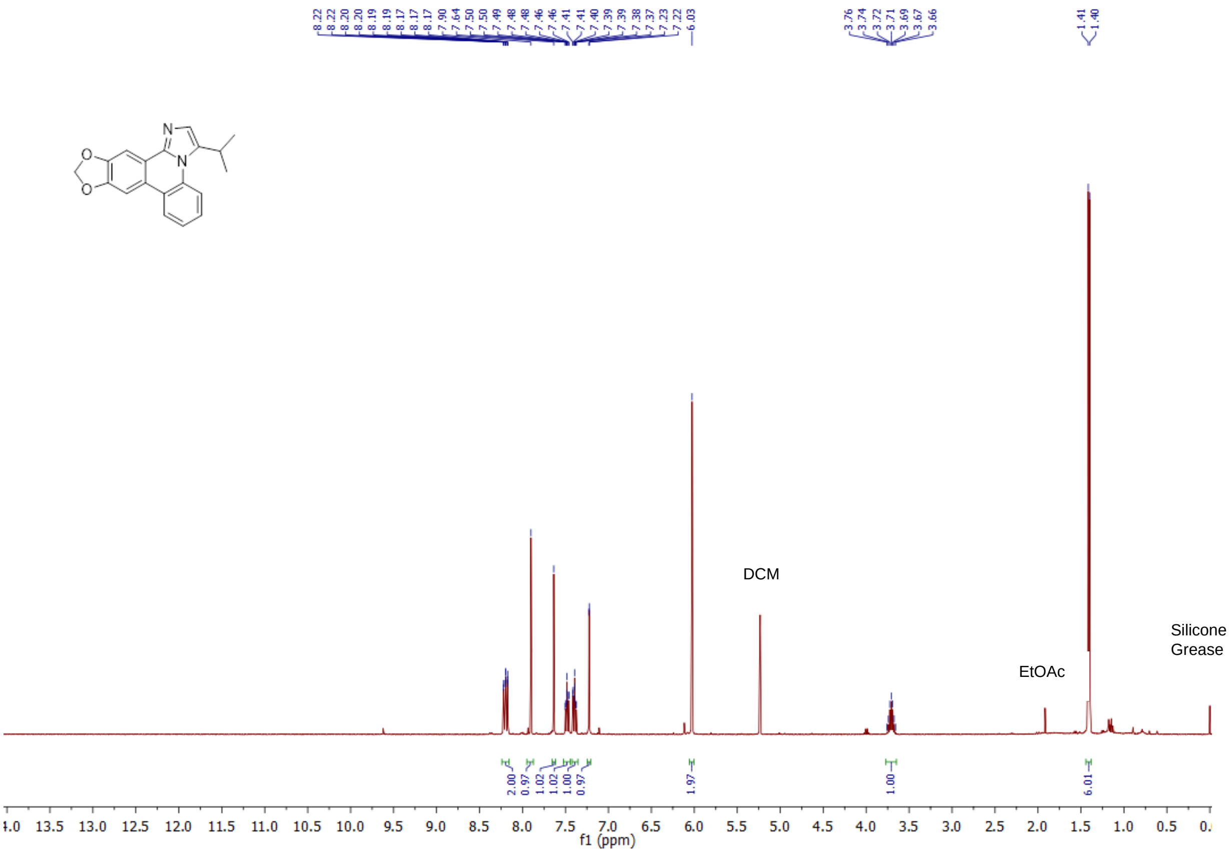

^{13}C NMR spectrum of compound 14 (101 MHz, methylene chloride-d_2)

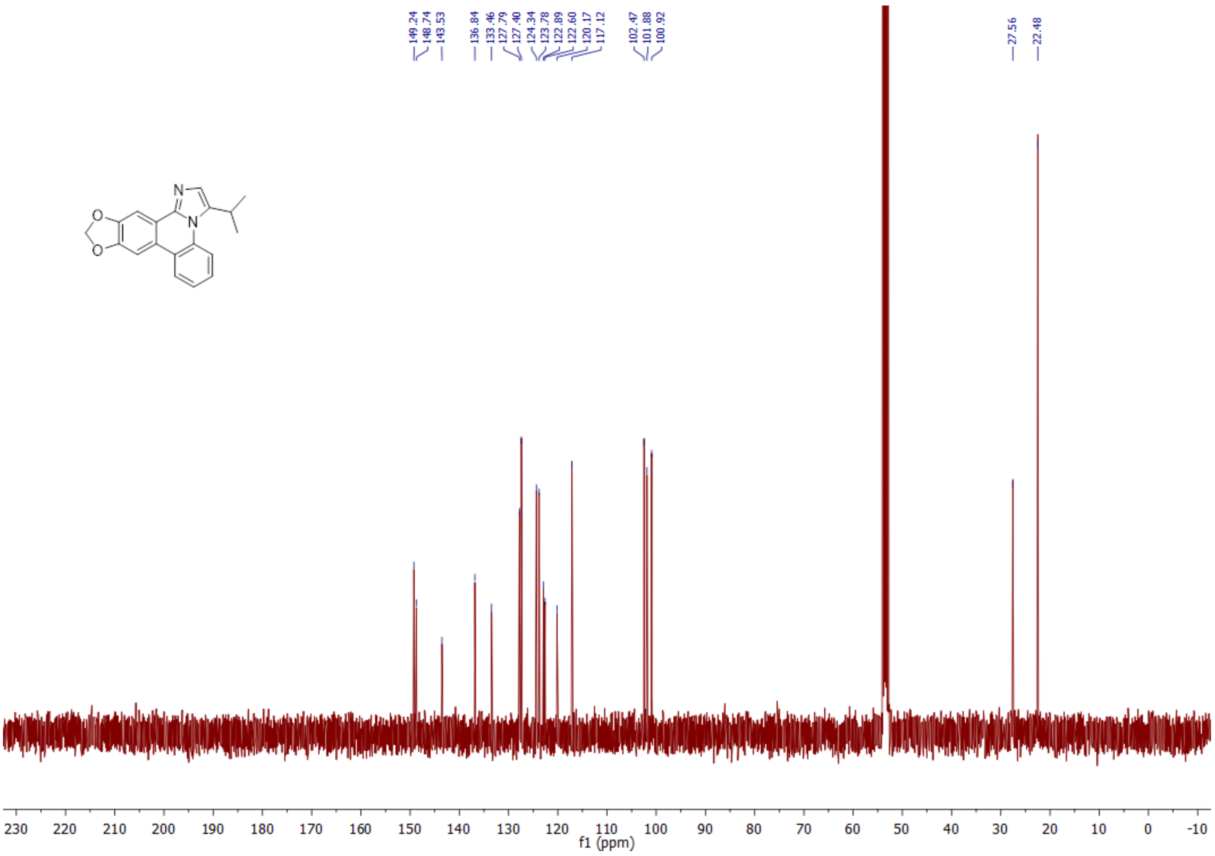

^{1}H NMR spectrum of compound 15 (400 MHz, methylene chloride-d_2)

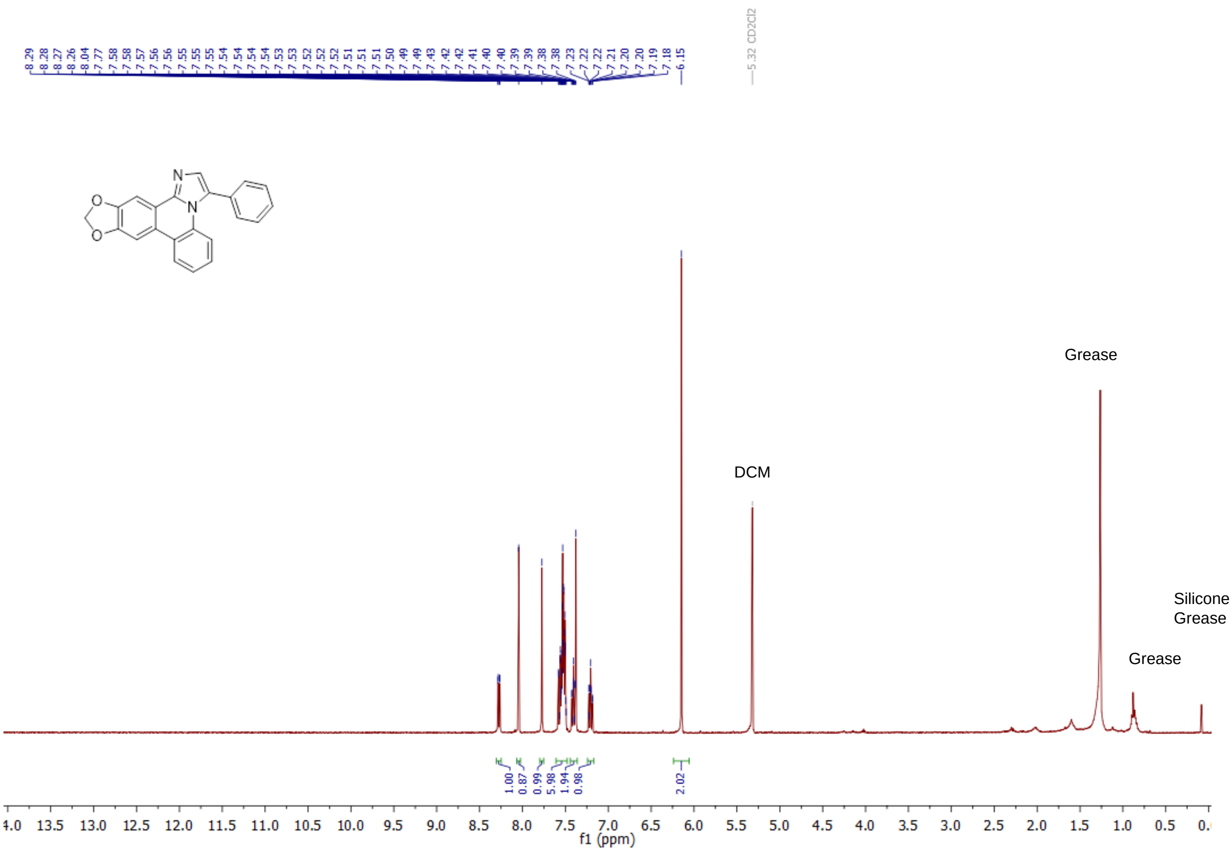

^{13}C NMR spectrum of compound 15 (101 MHz, methylene chloride-d_2)

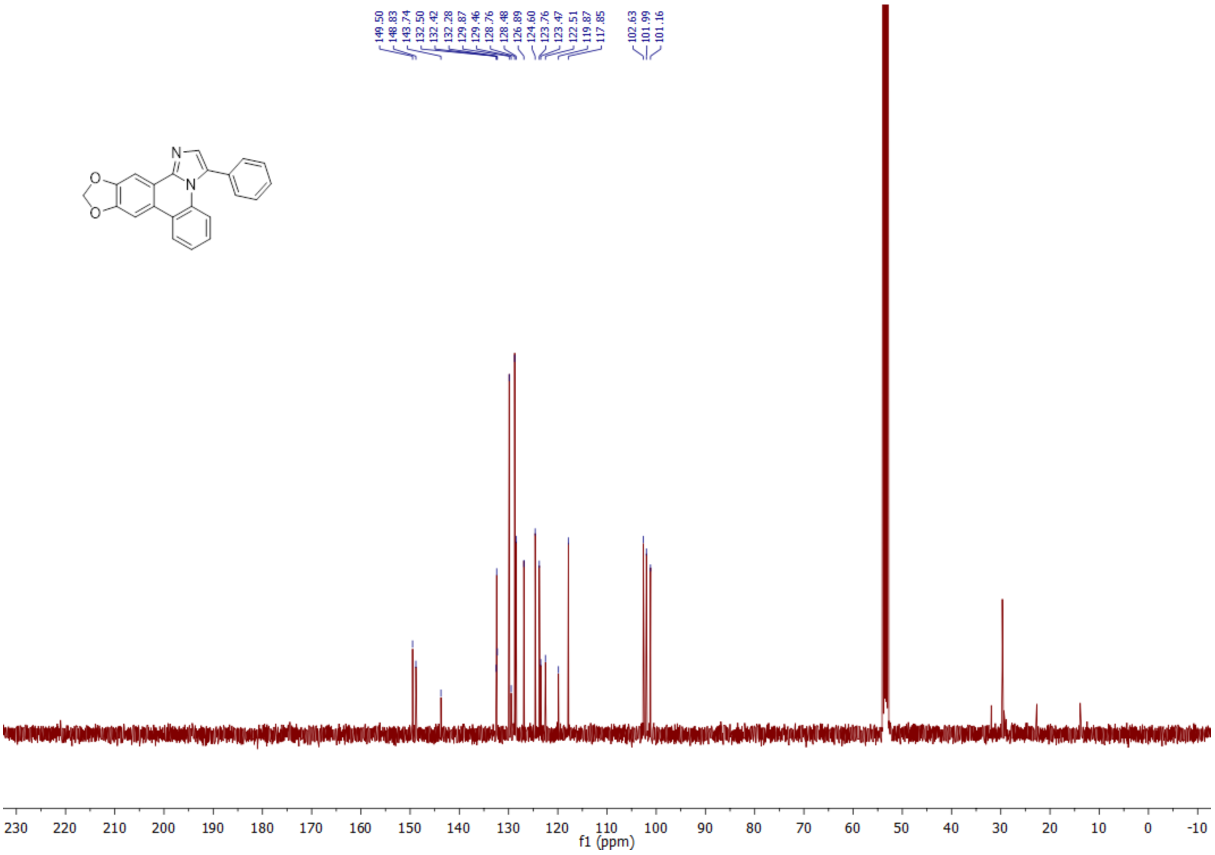

Table S1: List of IC_{50} values

Compound #	IC_{50} Jurkat [µM]	IC_{50} MCF7 [µM]	IC_{50} HL-60 [µM]	IC_{50} CEM [µM]	IC_{50} HUVEC [µM]	IC_{50} MCF10A [µM]
**Zeph A**	11.7	145	n.a.	19.5	50.4	10.4
**15**	26.8	79.4	/	/	/	
**14**	19.3	58.8	/	/	/	
**13**	9.6	32.7	27.3	12.6	72.6	16.3
**12**	41.06	69.4	55.8	51.3	56.6	35.0
**11**	11.8	33.7	14.2	10.3	67.1	18.6
**10**	28.2	120	/	/	/	
**9**	13.4	47.5	/	/	/	

n.a. = no activity; / = not tested
